# Influence of the Synthesis and Crystallization Processes on the Cation Distribution in a Series of Multivariate Rare-Earth Metal–Organic Frameworks and Their Magnetic Characterization

**DOI:** 10.1021/acs.chemmater.2c01481

**Published:** 2022-07-25

**Authors:** Raluca Loredana Vasile, Agustín Alejandro Godoy, Inés Puente Orench, Norbert M. Nemes, Víctor A. de la Peña O’Shea, Enrique Gutiérrez-Puebla, Jose Luis Martínez, M. Ángeles Monge, Felipe Gándara

**Affiliations:** † Materials Science Institute of Madrid—Spanish National Research Council (ICMM-CSIC), Calle Sor Juana Inés de la Cruz 3, 28049 Madrid, Spain; ‡ Instituto de Investigación en Tecnología Química (INTEQUI-CONICET), Universidad Nacional de San Luis, Alte. Brown 1450, D5700HGC San Luis, Argentina; § Institut Laue Langevin, 71 Avenue des Martyrs, Grenoble 38042, France; ∥ Instituto de Nanociencia y Materiales de Aragón (INMA-CSIC), Calle Pedro Cerbuna 12, 50009 Zaragoza, Spain; ⊥ Departamento de Física de Materiales, Facultad Físicas, Universidad Complutense de Madrid, E-28040 Madrid, Spain; # Photoactivated Processes Unit IMDEA Energy Institute, Móstoles Technology Park, Avenida Ramón de la Sagra 3, Móstoles, Madrid 28935, Spain

## Abstract

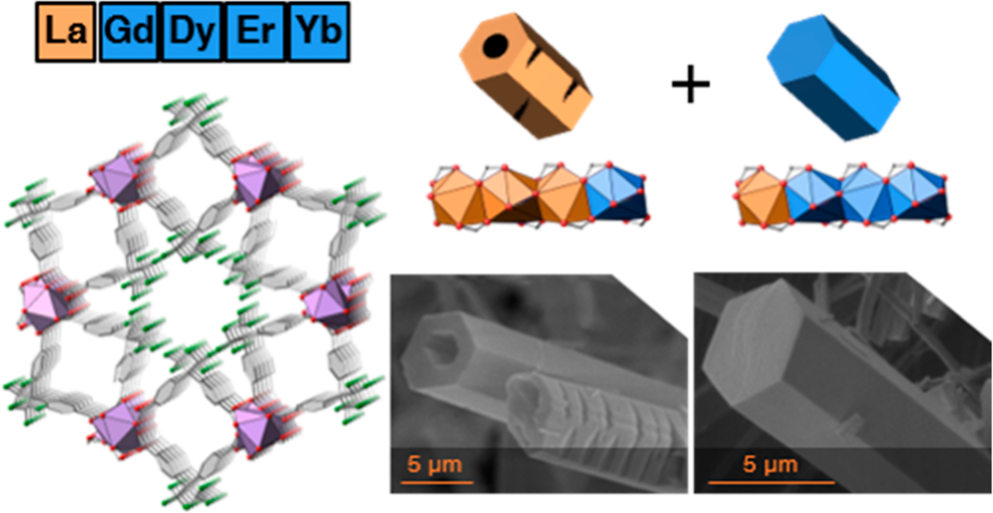

The incorporation of multiple metal atoms in multivariate metal–organic frameworks is typically carried out through a one-pot synthesis procedure that involves the simultaneous reaction of the selected elements with the organic linkers. In order to attain control over the distribution of the elements and to be able to produce materials with controllable metal combinations, it is required to understand the synthetic and crystallization processes. In this work, we have completed a study with the RPF-4 MOF family, which is made of various rare-earth elements, to investigate and determine how the different initial combinations of metal cations result in different atomic distributions in the obtained materials. Thus, we have found that for equimolar combinations involving lanthanum and another rare-earth element, such as ytterbium, gadolinium, or dysprosium, a compositional segregation takes place in the products, resulting in crystals with different compositions. On the contrary, binary combinations of ytterbium, gadolinium, erbium, and dysprosium result in homogeneous distributions. This dissimilar behavior is ascribed to differences in the crystallization pathways through which the MOF is formed. Along with the synthetic and crystallization study and considering the structural features of this MOF family, we also disclose here a comprehensive characterization of the magnetic properties of the compounds and the heat capacity behavior under different external magnetic fields.

## Introduction

Multivariate metal–organic frameworks (MTV-MOFs) are a class of MOFs that incorporate in the same structure multiple different functional groups^1−3^ or various metal elements,^4,5^ which occupy topologically equivalent positions in the framework without altering the network connectivity. In the case of multi-metal MTV-MOFs, different metal elements are distributed in such a way that the connectivity of the inorganic secondary building unit (SBU) of the MOF remains unaltered. Multi-metal MTV-MOFs are typically synthesized through the one-pot addition of the selected metal salts along with the organic linkers under conditions that allow the incorporation of the selected metal elements into the MOF backbone. Following this approach, a number of compounds have been synthesized with combinations of different numbers of metal elements, resulting in a modification or enhancement of the properties of the MOF, such as catalytic activity,^6−12^ gas sorption,^13,14^ biological activity/drug delivery,^15,16^ magnetism,^17^ energy storage,^18−21^ or optical activity.^22−24^

**Figure 1:**
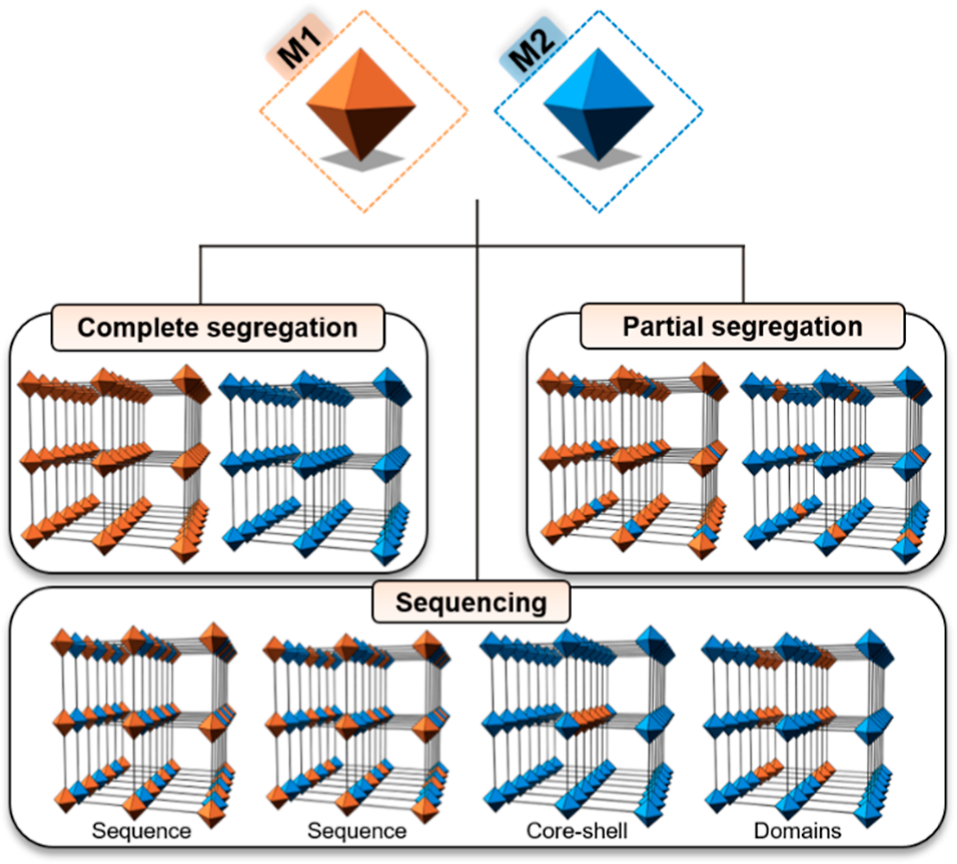
Combination of different metal cations results in multi-metal MTV MOFs as well as in the competitive formation of crystals with different metal combinations.

Among the different types of MOF families, those based on rod-shaped SBUs are particularly interesting for the formation of multi-metal MTV compounds because the metal cations are located in close vicinity through carboxylate bridges.^25,26^ This results in a high density of metal sites accessible through the MOF pores and provides the ability to form different atomic sequences by adjusting the metal cation combinations (Figure 1). MOF-74 is a representative example of a rod-shaped MOF. It was among the first ones to be reported in the multi-metal MTV form,^27,28^ and more recently, Yaghi et al. were able to map out different possible atomic sequences that are formed in multi-metal MTV-MOF-74 with the use of atom probe tomography, finding different types of arrangements depending on the employed reaction temperature.^29^ Nevertheless, with few exceptions,^30−34^ the majority of mixed-metal MTV-MOFs are composed of binary combinations of metal elements, and during their synthesis, it is assumed that the employed cations will be reacting in a similar way with the organic linkers. However, it is possible that during the MTV-MOF formation process, competing reactions take place simultaneously, potentially leading to the segregation of the metal cations, with the appearance of mixtures of different crystalline phases.^35^ In other cases, the different cations employed for the synthesis of a multi-metal MTV-MOF might behave differently and react at different rates with the organic linkers, for example, resulting in domain formations or core–shell type distributions.^36−39^

**Figure 2:**
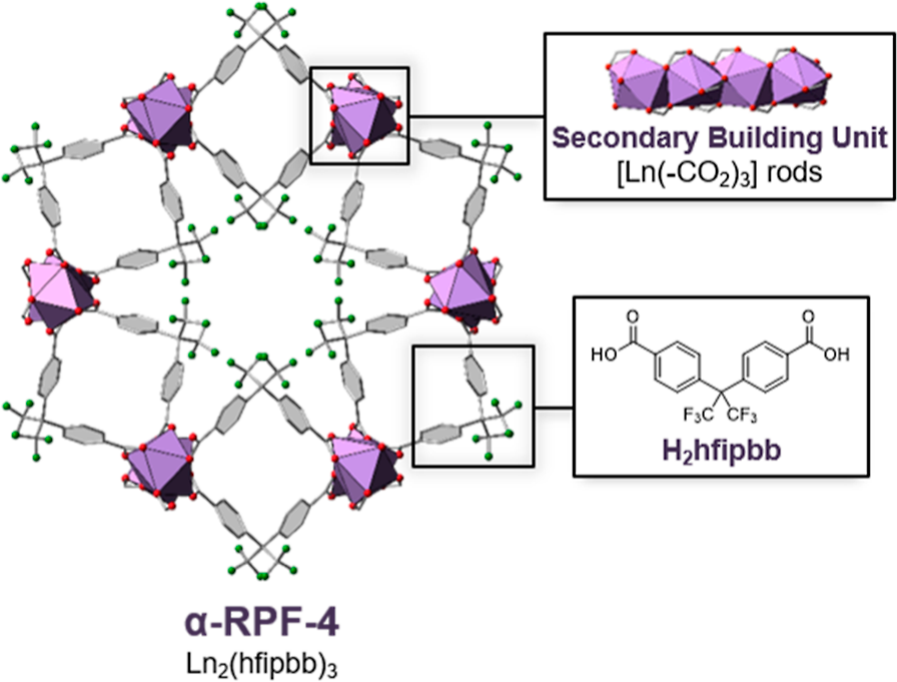
Crystal structure of RPF-4 consists of rod-shaped SBUs made of rare-earth cations connected by the organic linker H_2_hfipbb.

In our on-going investigation on multi-metal MOFs, we have studied the influence of the initial combination of different metal elements on the formation of different types of atomic sequences in an MOF family comprising a rod-shaped SBU. Thus, in previously reported works, we found that the initial metal combination not only results in the formation of different types of elemental sequences, and we also found that the crystallization mechanism might not be the same for different combinations of metal elements.^40−43^ Recently, we have turned our attention to another highly related MOF family based on the same (4,4′-hexafluoroisopropylidene)bis(benzoic acid) organic linker and rare-earth cations (Figure 2). In contrast to the previous example with transition-metal elements, this MOF family (first reported as RPF-4, rare-earth polymeric framework-4) can be obtained in a single-metal form with an array of lanthanide ions, which go from La to Yb.^44^ Lanthanide ions have a flexible coordination sphere due to their relative large size, and lanthanide-based MOFs containing rod-shaped SBUs have garnered additional interest due to the possible presence of one-dimensional magnetic interactions between the metal centers that are disposed at short distances, bridged by carboxylic groups.^45−47^ In the case of the RPF-4 structural type, the inorganic SBU comprises LnO_9_ polyhedra that share faces via three μ-O atoms, resulting in rare-earth cations at very short distances (e.g., La–La = 3.895 Å). Bearing these structural considerations in mind, in the present work, we have completed a comprehensive magnetic characterization of this MOF series, including single-metal and binary combinations, with interest in possible long-range magnetic order or magnetocaloric effects.

**Scheme 1:**
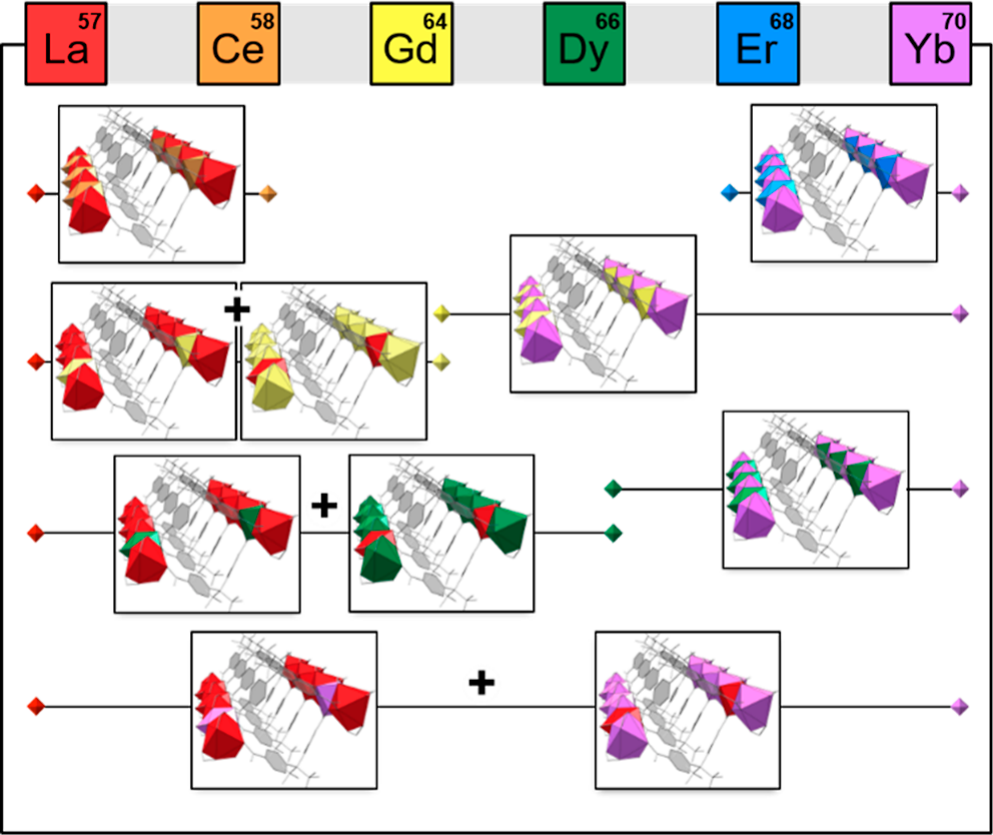
Depending on the Element of Choice, the Combination of Different Metal Cations in the RPF-4 Family Results in Multi-Metal MTV MOFs as Well as in the Competitive Formation of Crystals with Different Metal Combinations

From the synthetic perspective, the selection of RPF-4 allows us to further investigate the role of the initial metal combinations in the MOF crystallization process. Thus, we have studied the incorporation rates of the selected rare-earth cations, finding that although the resulting MOF topology remains unaltered, their formation follows different crystallization pathways, which are governed by the metal ions present in the synthesis medium. Most importantly, we found that certain combinations result in a competitive formation of isoreticular crystals with well-differentiated metal ratios in a same reaction batch (Scheme 1), even though the overall output appears to be homogeneous. Nonetheless, the introduction of different metal cations at desired ratios is accomplished for a number of rare-earth combinations, as evidenced by multiple of techniques, including neutron powder diffraction (NPD). These findings illustrate the relevance of understanding the crystallization process of multi-metal MTV-MOFs to acquire a higher control of the formation of atomic sequences.

## Experimental Details

All reagents and solvents employed were commercially available and used as received without further purification: 4,4′-(hexafluoroisopropylidene)bis(benzoic acid) (H_2_hfipbb) (>98%, TCI); lanthanum nitrate hexahydrate, La(NO_3_)_3_ × 6H_2_O (99.9%, Alfa Aesar); cerium nitrate hexahydrate, Ce(NO_3_)_3_ × 6H_2_O (99%, Aldrich); gadolinium nitrate hexahydrate, Gd(NO_3_)_3_ × 6H_2_O (99.9%, Strem Chemicals); dysprosium nitrate hexahydrate, Dy(NO_3_)_3_ × 6H_2_O (99.9%, Strem Chemicals); holmium nitrate hydrate, Ho(NO_3_)_3_ × *n*H_2_O (99.9%, Strem Chemicals); erbium nitrate pentahydrate (99.99%, Alfa Aesar), Er(NO_3_)_3_ × 5H_2_O ; ytterbium nitrate pentahydrate, Yb(NO_3_)_3_ × 5H_2_O (99.9%, Strem Chemicals); ethanol absolute (Scharlau); and acetone (99.6%, Labkem).

### Synthesis of Single-Metal MOFs

0.115 mmol of Ln(NO_3_)_3_ × *n*H_2_O (Ln = La, Ce, Gd, Dy, Ho, Er, and Yb) and 0.176 mmol of H_2_hfipbb were dissolved in a solvent mixture of 7.5 mL of absolute ethanol and 5 mL of water. The mixture was placed in a 50 mL Teflon-lined steel autoclave and heated overnight in an oven at 160 °C. After cooling to room temperature, the crystals were filtered and washed with water and acetone.

### Synthesis of Multi-Metal MTV-MOFs

All the multi-metal samples were obtained following the same synthetic strategy as for the single-metal MOFs. The amount of metal salt was adjusted according to every combination. All the quantities of metal salts used in all of the synthesis are listed in Table S1. To illustrate how each combination was obtained, an example is given as follows: for the synthesis of the La–Yb 1:9 combination, La(NO_3_)_3_ × 6H_2_O (0.0115 mmol, 4.98 mg), Yb(NO_3_)_3_ × 5H_2_O (0.1035 mmol, 46.53 mg), and H_2_hfipbb (0.176 mmol, 70.45 mg) were dissolved in 7.5 mL of absolute ethanol and 5 mL of water. The mixture was placed in a Teflon-lined steel autoclave and heated overnight in an oven at 160 °C. After cooling to room temperature, the crystals were filtered and washed with water and acetone.

### Powder X-ray Diffraction

Powder X-ray Diffraction (PXRD) patterns were collected with a Bruker D8 DaVinci diffractometer equipped with a Ni-filtered CuKα radiation tube (Kα1 = 1.5406 Å, Kα2 = 1.5444 Å, and Kα1/Kα2 = 0.5) operated at a voltage of 40 kV and at a current of 40 mA. Each experiment was recorded with an exposure time of 0.1 s per step and a step size of 0.02°. The samples were prepared by placing a small amount of a suspension of crystals in acetone on a glass sample carrier, forming a thin layer in the center.

### Scanning Electron Microscopy and Energy-Dispersive X-ray Spectroscopy

Scanning electron microscopy (SEM) studies were conducted at the Materials Science Institute of Madrid, with a FE-SEM FEI Nova NANOSEM 230 microscope equipped with an Everhard-Thornley ETD detector and an operating voltage between 5 and 15 kV. Some of the studies were occasionally performed at the Interdepartmental Research Service (Sidi) from the Universidad Autónoma de Madrid (Spain) with a Hitachi S-3000 N microscope equipped with a Bruker Quantax EDS XFlash 6I30 analyzer. The samples were prepared by placing the crystals on a double-sided adhesive conductive carbon tape that was attached to a flat aluminum sample holder, which was metallized with a gold layer of 127.5 Å with a Leica EM ACE200 sputter. EDS microanalysis were performed with an EDAX Apollo 10–300 mm detector. Several points of the crystals were recorded, generally from the basal planes and the body of the crystal. The ratio between the metals was calculated following the formula:
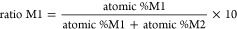


### Total-Reflection X-ray Fluorescence

Total-reflection X-ray fluorescence (TXRF) spectrometry analysis was performed at the Interdepartmental Research Service (Sidi) from the Universidad Autónoma de Madrid (Spain) with a Bruker S2 PicoFox spectrometer equipped with a Peltier-cooled XFlash Silicon Drift detector operated at a voltage of 50 kV and a current of 600 μA.

### Neutron Powder Diffraction

NPD data were acquired with D1B instrument of the Institut Laue Langevin (France). For the room temperature measurements, samples were contained in 5 mm cylindrical vanadium cans, while for the sub-kelvin experiments, a 6 mm cylindrical copper container was used. All data sets were recorded with a monochromatic neutron wavelength of 2.5260 Å. The peak broadening contribution of the instrument was determined for the refinement of the standard Na_2_Ca_3_Al_2_F_14_. The main contribution of parasitic diffraction peaks arising from the environment of the sample is removed by a radial oscillating collimator.

### Magnetization Measurements

DC magnetization measurements were performed using a SQUID magnetometer MPMS-5 S from Quantum Design (San Diego, USA) in a temperature range from 1.8 to 400 K and a range of magnetic fields up to 5 T. The AC magnetic susceptibility measurements were performed on a multipurpose platform (physical property measurement system, PPMS) from Quantum Design (San Diego, USA) in a temperature range from 1.8 to 400 K, with an excitation modulate field of 1–10 Oe in amplitude and the frequency range from 10 Hz to 10 kHz. Also, we were able to measure the AC susceptibility under a superimposed DC field in the range from 0 to 9 T.

### Thermal Measurements

The heat capacity measurements were performed in the PPMS platform (mentioned above) with the heat pulse method. The sample was attached (with Apiezon N-grease) to a small sapphire plate, which has a calibrated heater and a Cernox thermometer in the bottom part. By a heat pulse and the measurement of the temperature relaxation, the heat capacity is calculated under the approach of one or two relaxation times. A previous measurement of the heat capacity produced by the grease addenda was made before each measurement. The temperature range covered in these measurements was from 1.8 to 300 K, and the applied external magnetic field extended from 0 to 9 T.

## Results and Discussion

The first step of our study was to synthesize and characterize various samples of single-metal RPF-4 with an array of lanthanide ions of different atomic radii (La^3+^, Ce^3+^, Nd^3+^, Eu^3+^, Gd^3+^, Tb^3+^, Dy^3+^, Ho^3+^, Er^3+^, and Yb^3+^). PXRD measurements (Figure S1) confirmed that all single-metal samples display the same crystal structure, which belongs to the orthorhombic *Pnan* space group, and it consists of rod-shaped SBUs with nine-coordinated Ln^3+^ cations connected through the organic linkers to create channels that run parallel to the SBUs along the crystallographic *a* axis (Figure 2).

**Figure 3:**
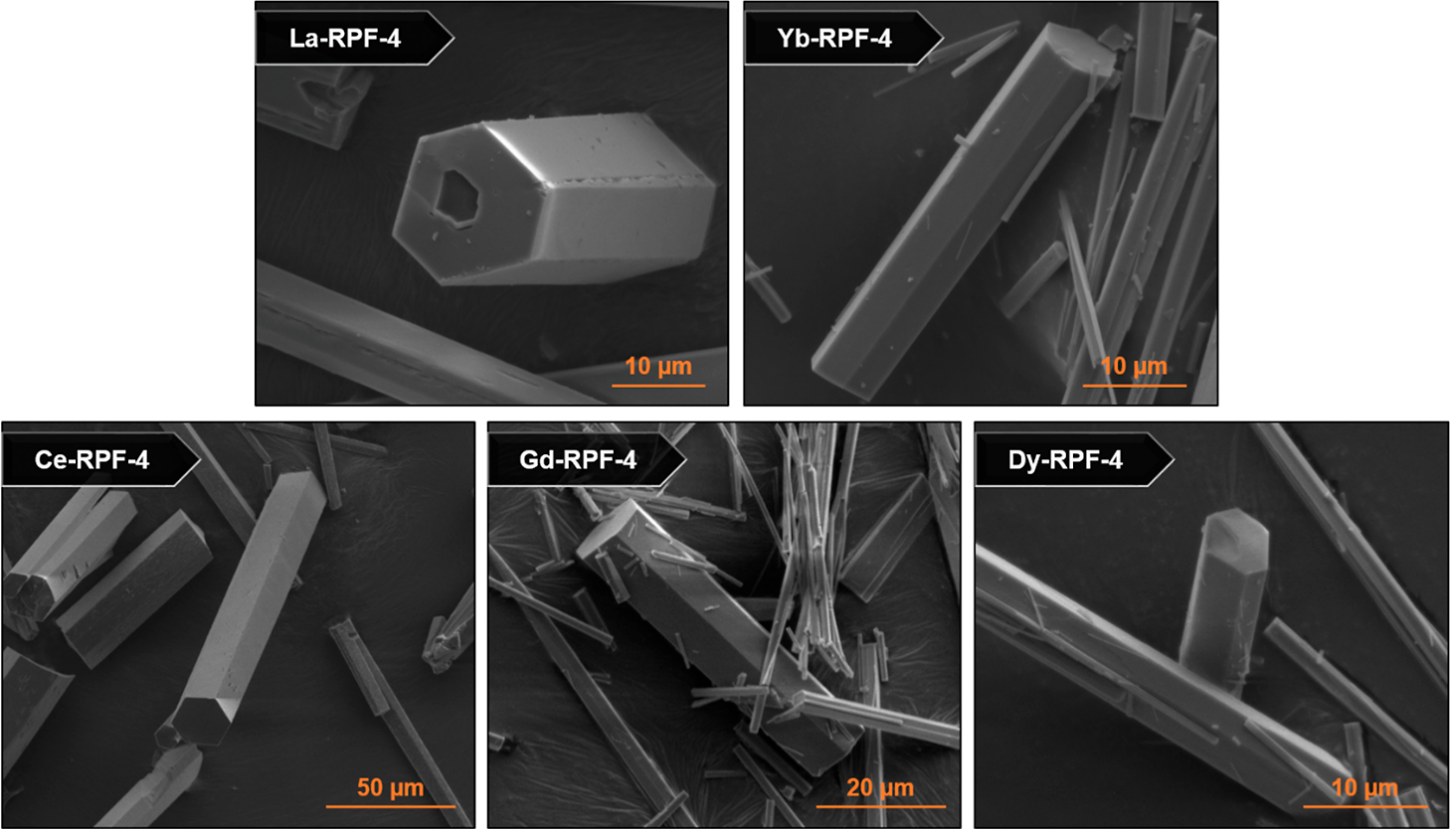
SEM images of the single-metal RPF-4 samples.

Subsequently, we completed the SEM study for the samples to examine their crystal morphologies in order to establish whether MOF synthesis with each cation follows the same crystallization mechanism, finding differences in the morphological features depending on the selected metal cation. Figure 3 shows SEM images of the single-metal samples, where it can be seen that the crystals of La-RPF-4 exhibit an uneven texture with the presence of holes. In contrast, samples corresponding to RPF-4 synthesized with any of the other cations showed smooth crystals with well-defined faces and no obvious defects. The presence of hollow crystals, with holes in their inner part indicates that the crystallization of La-RPF-4 follows an Ostwald ripening mechanism^48,49^ based on initial nucleation events and subsequent dissolution of the inner part of the crystal, followed by growing of the external faces. This is indicative of a different crystallization pathway for La-RPF-4 versus the other members of the family, resulting in particular morphologies with the presence of visible indents, cracks, and holes in the crystals. We previously observed a similar mechanism in the growing of related MTV Zn–Co samples,^41^ where the initial nucleation was ascribed to the formation of nuclei with only zinc in the SBUs, which were later re-dissolved to continue growing with the incorporation of both metal elements. However, we did not observe a similar behavior for the single-metal MOFs.

**Figure 4:**
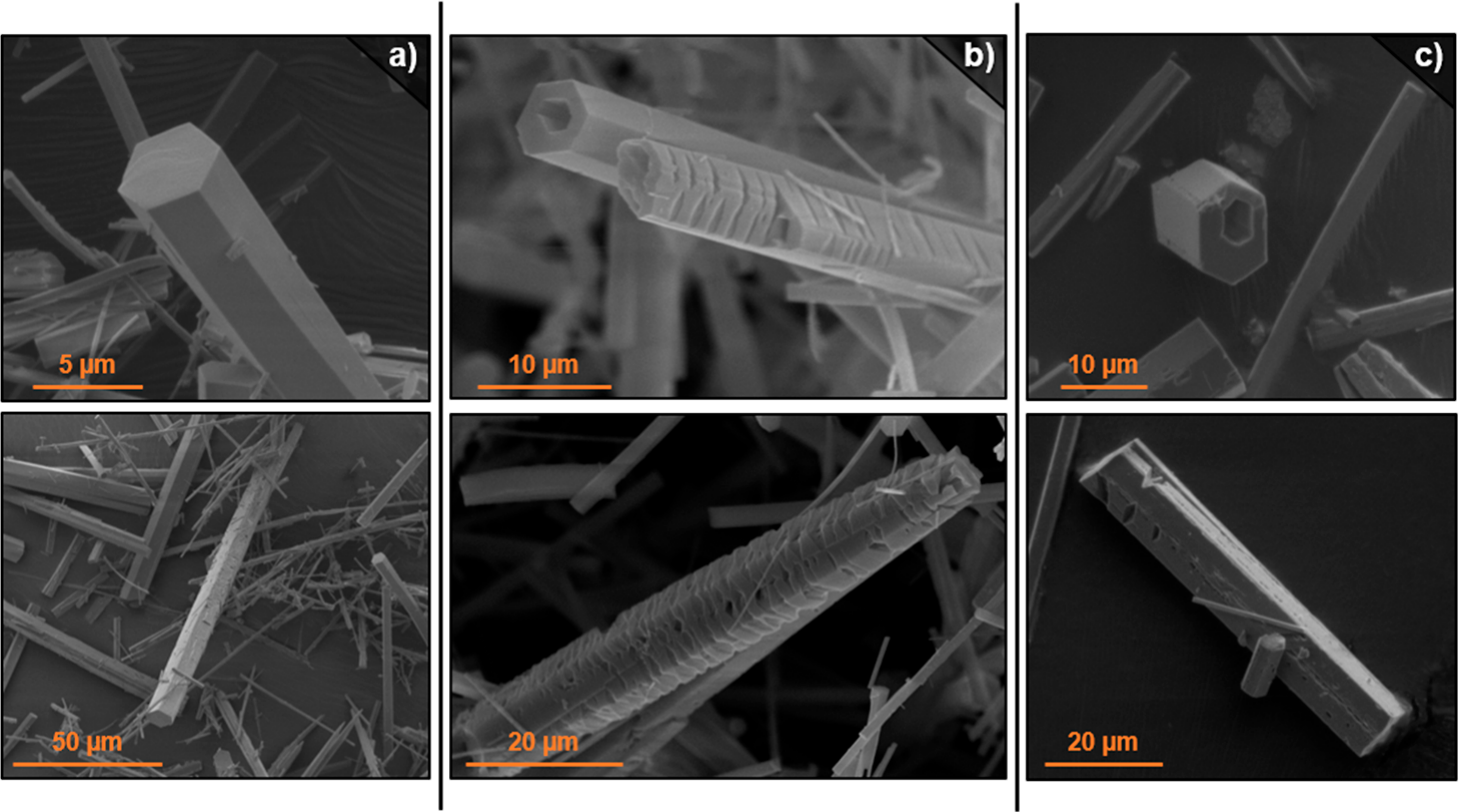
SEM images of crystals corresponding to LaYb–RPF-4 combinations; (a) La–Yb 1:9; (b) La–Yb 1:1; and (c) La–Yb 9:1.

**Figure 5:**
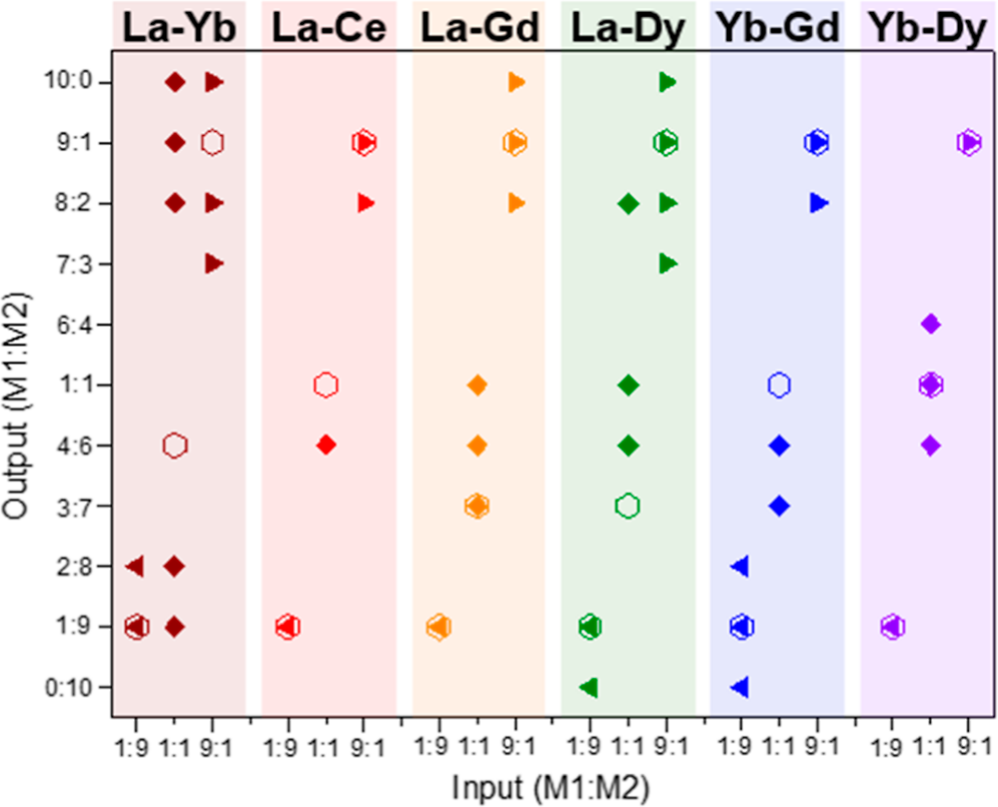
Comparation between the input and the output metal ratio (EDS values found in the samples) of all the combinations that were synthesized in 1 day; the hollow hexagons indicate the composition of the bulk according to TXRF results.

Binary combinations: Upon evidencing the differences in the crystallization process along the various single-metal RPF-4 samples, we continued our study with binary combinations of rare-earth elements to evaluate how they possibly impact the formation of the resulting multi-metal MTV-MOFs and the incorporation of the different elements into the frameworks. For all metal combinations, we selected three different initial molar ratios, 1:9, 1:1, and 9:1, while keeping constant the metal/linker ratio and the amount of solvent. These metal ratios were selected to investigate the different scenarios where either one of the elements is in a significantly higher concentration than the second one and possibly directing the MOF initial formation, or both of them are in the same concentration, which can lead to an incorporation competition during the crystallization. PXRD patterns of all the samples demonstrated that in all cases, the same RPF-4 was formed as a pure crystalline phase (Figures S2–S8). We then proceeded with the SEM study, including the elemental composition analysis of the crystals with EDS measurements. Accordingly, for each sample, multiple microanalyses were made in several areas of various individual specimens throughout both the basal plane and the body of the crystals. Our starting point was the combination of lanthanum and ytterbium to investigate the adaptability of the structure to withstand the incorporation of elements with differently enough atomic radius. Synthesis reactions carried out with a La–Yb 9:1 initial ratio showed the formation of crystals with features equivalent to the ones observed for single-metal La-RPF-4, such as inner holes and rough surfaces (Figure 4). EDS analysis demonstrated that ytterbium is incorporated into the crystals in the same ratio as the input. Conversely, for reactions carried out with La–Yb 1:9 ratios, the morphology of the crystals corresponds to the one observed for Yb-RPF-4, exhibiting well-defined smooth faces. These observations indicate that MTV systems can be synthesized with these two cations, avoiding the formation of individual single-metal phases. It is also implied that the crystallization is governed by the element present in a higher amount while allowing the introduction of the second one at a small concentration. However, when increasing the amount of ytterbium in reactions with La–Yb 8:2 7:3 or 6:4 initial ratios, the EDS analyses consistently showed that the resulting MOF stoichiometries did not follow the same trend as that of the input. Instead, the output ratios were close to the same 9:1 values (see Figure 5). This compositional bias was also observed for the corresponding reactions where ytterbium was added in a higher amount, namely, La–Yb 2:8, 3:7, and 4:6. In all cases, the morphological features of the crystals were equivalent to the ones displayed by the corresponding single-metal samples. Thus, for those reactions where a higher amount of lanthanum was added, the crystals clearly showed the presence of inner holes, while for those where ytterbium was in a higher concentration, all crystals were smooth with flawless faces. Most interestingly, the experiment carried out with an equimolar La–Yb starting ratio showed co-existence of both types of crystals with different features and compositional outputs. Thus, although the overall bulk composition obtained by TXRF analysis indicated a La–Yb 4:6 average output, the SEM study demonstrated that the formation of an MTV-MOF with a 1:1 ratio was not accomplished, and instead, crystals rich in lanthanum were formed along with others rich in ytterbium. Crystals with different features were clearly observed, in line with their distinct compositions.

On viewing these results, we wondered whether combinations of other rare-earth elements with closer atomic numbers would result in similar outputs or, on the contrary, it would be possible to obtain multi-metal MTV-MOFs with equimolar combinations. Therefore, we completed the corresponding synthesis experiments for La–Ce systems, with initial ratios of 1:9, 1:1, and 9:1. Similarly to the previous case, when one of the metal elements was in excess with respect to the other one, the resulting outputs were in harmony with that initially added. On the contrary, the experiment carried out with an equimolar combination showed that it is feasible to obtain crystals with a 1:1 ratio of La–Ce. Moreover, a homogeneous distribution of the cations was now apparent, as shown by the EDS microanalysis results, which were consistently found to be 1:1, within the experimental error. Regarding the morphology of the crystals, there was no obvious presence of holes or cracks on the surface.

**Figure 6:**
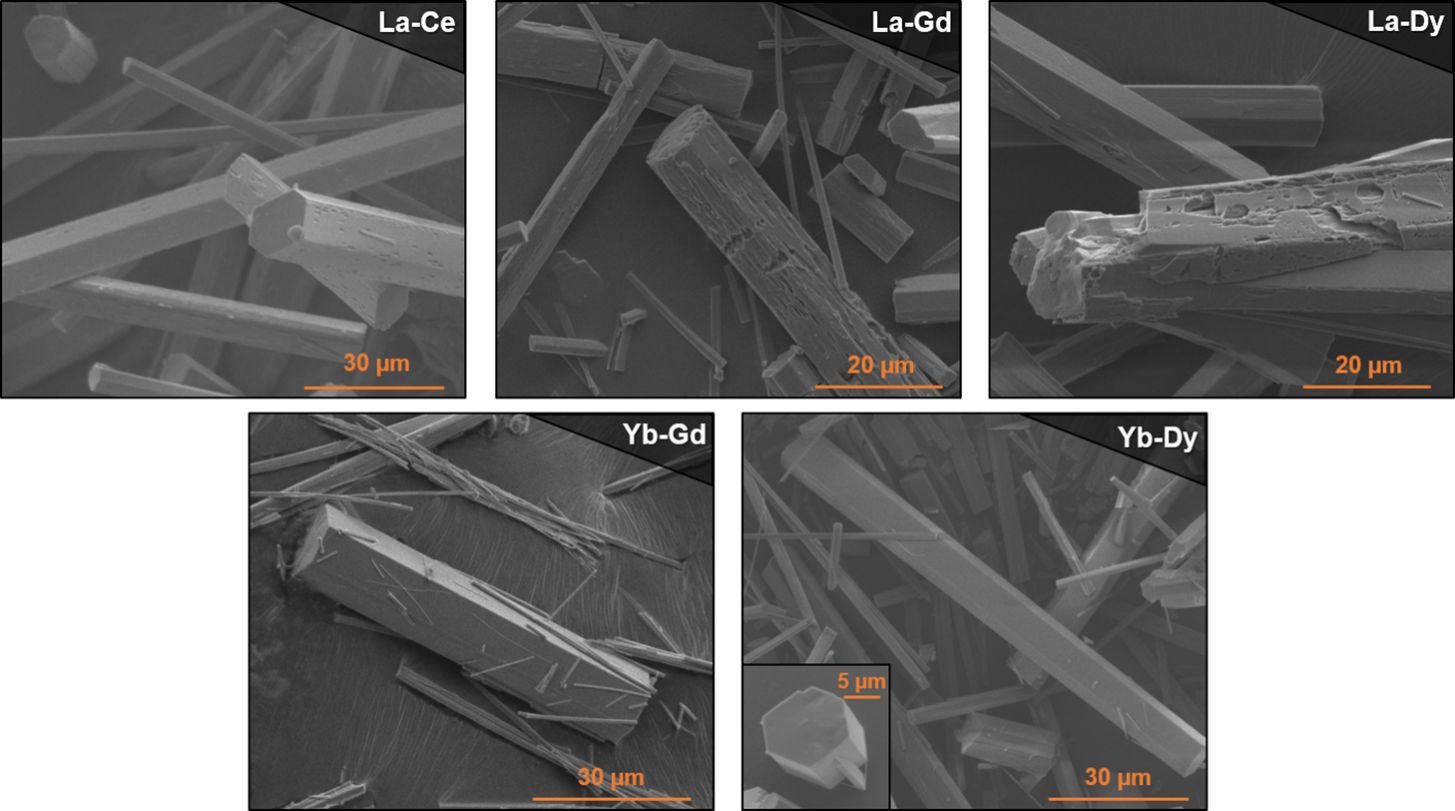
SEM images of crystals corresponding to equimolar RPF-4 combinations.

Moving on to combinations of lanthanum with another rare-earth element further apart in the series, we completed the synthesis experiments with La–Gd and La–Dy combinations. In both cases, the results for the non-equimolar initial mixtures are congruent with the behavior observed for the previous combinations. Slight differences in the morphology were found, though. Thus, the samples prepared with lanthanum as the cation in a higher concentration in the presence of dysprosium or gadolinium displayed indents as the main morphological defects. Concerning the equimolar ratio, in a fashion similar to that of the La–Yb combination, there was a coexistence of two types of crystals, and the distribution of the metal elements was not homogeneous, as demonstrated by the EDS analysis (Figure 6 and Table 1). Thus, for La–Gd, the average output ratio was found to be 3:7. However, an uneven distribution of metal ratios among different crystals was evident, finding a 4:6 distribution in some of the measurements. Meanwhile, for La–Dy combinations, the average ratio was found to be 6:4 according to TXRF bulk analysis, and now the EDS observed ratios for individual crystals ranged over La–Dy 8:2, 1:1, and 4:6.

**Table 1:** Starting Molar Ratio of Each RPF-4 Combination and EDS and TXRF Data for the Output

			metal ratio for 1 day synthesis Ln_2_(hfipbb)_3_		metal ratio for 3 day synthesis Ln_2_(hfipbb)_3_	
combination	initial molar ratio		EDS average	TXRF bulk	EDS average	TXRF bulk
La–Yb	1	9	La_0.2_Yb_1.8_	La_0.2_Yb_1.8_	La_0.1_Yb_1.9_	
					La_0.6_Yb_1.4_	
	1	1	La_0.4_Yb_1.6_	La_0.8_Yb_1.2_	La_0.4_Yb_1.6_	La_1.0_Yb_1.0_
			La_1.8_Yb_0.2_		La_1.2_Yb_0.8_	
	9	1	La_1.8_Yb_0.2_	La_1.8_Yb_0.2_	La_1.8_Yb_0.2_	
La–Ce	1	9	La_0.2_Ce_1.8_	La_0.2_Ce_1.8_	La_0.2_Ce_1.8_	
	1	1	La_0.8_Ce_1.2_	La_1.0_Ce_1.0_	La_1.0_Ce_1.0_	La_1.0_Ce_1.0_
	9	1	La_0.2_Ce_1.8_	La_0.2_Ce_1.8_	La_1.6_Ce_0.4_	
La–Gd	1	9	La_0.2_Gd_1.8_	La_0.2_Gd_1.8_	La_0.2_Gd_1.8_	
	1	1	La_0.6_Gd_1.4_	La_0.6_Gd_1.4_	La_0.8_Gd_1.2_	La_1.0_Gd_1.0_
			La_0.8_Gd_1.2_		La_1.4_Gd_0.6_	
	9	1	La_1.8_Gd_0.2_	La_1.8_Gd_0.2_	La_1.8_Gd_0.2_	
La–Dy	1	9	La_0.2_Dy_1.8_	La_0.1_Dy_1.9_	La_0.2_Dy_1.8_	
	1	1	La_0.8_Dy_1.2_	La_0.6_Dy_1.4_	La_0.6_Dy_1.4_	La_0.8_Dy_1.2_
			La_1.6_Dy_0.4_		La_1.6_Dy_0.4_	
	9	1	La_1.8_Dy_0.2_	La_1.8_Dy_0.2_	La_1.8_Dy_0.2_	
Yb–Gd	1	9	Yb_0.2_Gd_1.8_	Yb_0.2_Gd_1.8_	Yb_0.2_Gd_1.8_	
	1	1	Yb_0.6_Gd_1.4_	Yb_1.0_Gd_1.0_	Yb_0.6_Gd_1.4_	Yb_1.0_Gd_1.0_
					Yb_1.2_Gd_0.8_	
	9	1	Yb_1.8_Gd_0.2_	Yb_1.8_Gd_0.2_	Yb_1.8_Gd_0.2_	
Yb–Dy	1	9	Yb_0.2_Dy_1.8_	Yb_0.2_Dy_1.8_	Yb_0.2_Dy_1.8_	
	1	1	Yb_1.0_Dy_1.0_	Yb_1.0_Dy_1.0_	Yb_1.0_Dy_1.0_	Yb_1.0_Dy_1.0_
	9	1	Yb_1.8_Dy_0.2_	Yb_1.8_Dy_0.2_	Yb_1.8_Dy_0.2_	
Yb–Er	1	1			Yb_1.0_Er_1.0_	Yb_1.0_Er_1.0_

Bearing these results in mind, it is clear that there is a correlation among the distance between lanthanum and the combined element in the series and the ability to prepare the corresponding multi-metal MTV-MOFs with a homogeneous distribution of crystals with the same compositions. Thus, although MTV-MOFs are formed in all cases, a compositional segregation is clearly generated for certain combinations, which seems to be related to differences in the crystallization process, as suggested by the clear changes in the features of the crystals. In other binary combinations not involving lanthanum, we did not observe this effect. Thus, when mixing ytterbium with gadolinium or dysprosium in an equimolar amount, all the crystals displayed a smooth and flawless surface. However, while for Yb–Dy combinations, the output metal ratio was found to be coincident with the initially added, a compositional bias between input and output was observed for the Yb–Gd 1:1 initial combination, where the average output was found to be 3:7. Considering that there is no compositional segregation between crystals, this bias is probably related to kinetic effects, meaning that the incorporation rate of one of the metal elements into the MOF is faster than that of the other one.

**Figure 7:**
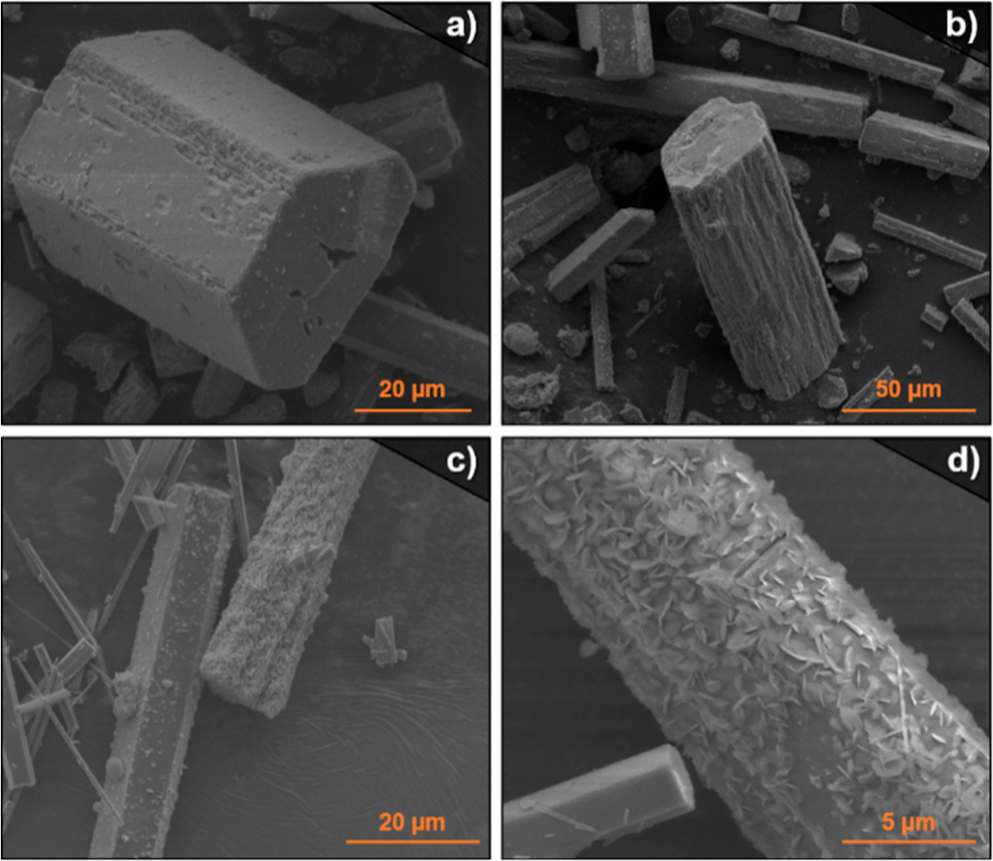
SEM images of the crystals obtained by combining single-metal La-RPF-4 and Yb(NO_3_)_3_ (a,b) or Yb-RPF-4 and La(NO_3_)_3_ (c,d).

To further investigate this point, we carried out synthesis experiments with the same initial metal ratios and combinations, but extending the reaction time from 1 day to 3 days. For the La–Yb 1:9 and 9:1 binary combinations, no evident changes were observed in crystal features or compositions, and the obtained samples maintained the same metal ratios as those initially added. For the sample prepared from an initial equimolar amount of metals, we now found the presence of crystals with new compositions not observed for the 1 day prepared sample. While a compositional segregation was still evident, crystals with an output metal ratio close to the input one, such as La–Yb 6:4 and 1:1, were formed after a 3 day synthesis (Table 1). A question arises whether these crystals resulted from new nucleation events or as result of partial redissolution and growing of those already formed after 1 day, comparable to an in situ trans-metalation process. To investigate this point, crystals of monometallic La- and Yb-RPF-4 were placed under the same synthetic conditions in the presence of the corresponding second element salt, finding particular behaviors in each case. Thus, when adding ytterbium nitrate to the La-RPF-4 crystals, the resulting samples contain both elements in the expected ratios (Figure S11), demonstrating that under these conditions, the trans-metalation process takes place. This is also consistent with the previous observations regarding the La-RPF-4 crystal features, indicative of an Ostwald ripening process, which should facilitate the insertion of ytterbium into the framework. As opposed to this, we found that when lanthanum nitrate is added to the Yb-RPF-4 sample, the insertion of lanthanum happens only on the surface of the crystals. Thus, these Yb-RPF-4 crystals appear to be covered with new particles, as shown in Figure 7. The EDS analysis (Figure S12) confirms the presence of lanthanum in these new particles, which were not dissolved after washing the samples, ruling out the possibility of them being lanthanum nitrate salt. Moreover, the PXRD analysis (Figure S9) did not show the presence of any other different crystalline phase, indicating that new RPF-4 nucleation is happening on the surface, possibly emerging from a partial surface reconstruction process, similar to the one that we previously observed for other rare-earth MOF,^50^ but excluding the possibility of a trans-metalation process along the crystals.

Increasing the reaction time to 3 days did not seem to have an obvious effect on the output metal ratios for combinations of lanthanum with cerium or dysprosium or ytterbium with dysprosium. For the La–Dy 1:1 initial combination, crystals with a non-homogeneous compositional distribution with comparable values to those of 1 day were observed. As for La–Gd combinations, differences were found in the output ratios for the samples prepared from the initial 1:1 input as compared to the 1 day preparations. While compositional segregation is still observed, the output ratio shows a larger average lanthanum content. For the Yb–Gd 1:1 samples, the increase in the reaction time to 3 days allows the obtaining of crystals with the same input and output metal ratios, eliminating the compositional bias seen for the 1 day prepared samples, illustrating the important effect of incorporation -kinetic rates of different metal elements on the obtaining of MTV multi-metal MOFs.

**Figure 8:**
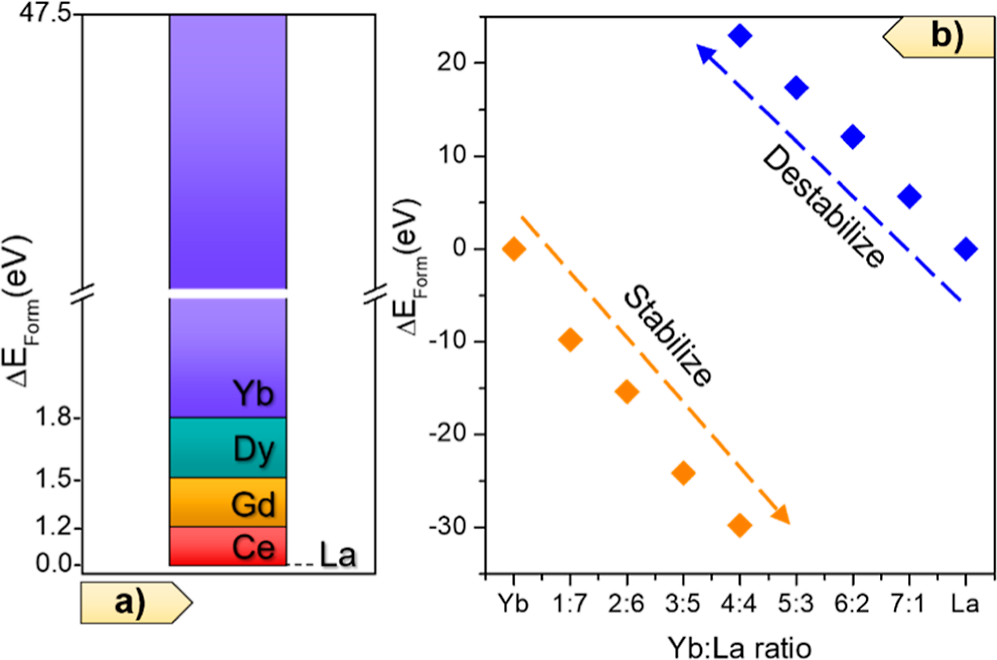
Relative values of formation energy for (a) various single-metal RPF-4 structures, referenced to La-RPF-4, and for (b) binary La–Yb samples. Here, the calculations were computed by starting from the corresponding single-metal structures and by replacing an increasing number of metal atom sites.

On viewing these results, it appears evident that there is a barrier for the formation of certain binary combinations. Thus, the fact that equimolar mixtures of particular element combinations result in the formation of crystals with different compositions, such as the case of the La–Yb system, indicates that there is a barrier in the formation of SBUs with atomic sequences consisting of alternating lanthanum and ytterbium atoms, leading to the obtaining of compositionally segregated crystals. This compositional segregation is associated with differences in the crystallization mechanism, involving partial redissolution during the reaction. However, it is also possible that the formation of certain binary combinations is energetically impeded. To further investigate this point, we completed DFT-based calculations to compute the relative formation energy of the lanthanide systems investigated here. Single-metal compounds show a formation energy difference between 1.2 and 1.8 eV, except for Yb-RPF-4, which is the one with the largest energy (Figure 8a). For a deeper understanding of the formation of bimetal systems, we have selected La–Yb RPF-4. To do this, we computed the formation energy of La-RPF-4 structures by replacing an increasing number (1 to 4) of La metal sites in the unit cell by Yb ions. Conversely, we computed the energy of Yb-RPF-4 where the metal sites are replaced by La ions. In both cases, we found a linear trend but with opposite results. While the introduction of La atoms in Yb-RPF-4 leads to a stabilization of the structure, the inclusion of Yb destabilizes the La-RPF-4 system (Figure 8b). These calculations in comparison with experimental results (Figure 5 and Table 1) indicate that even when La-RPF-4 is the most thermodynamically stable phase, kinetic factors play a crucial role in the bimetal MTV-MOF formation. Nevertheless, these results also evidence that different crystallization mechanisms co-exist during the MOF synthesis, with thermodynamic control for reactions with large amounts of lanthanum and kinetic control for the case of ytterbium.

**Figure 9:**
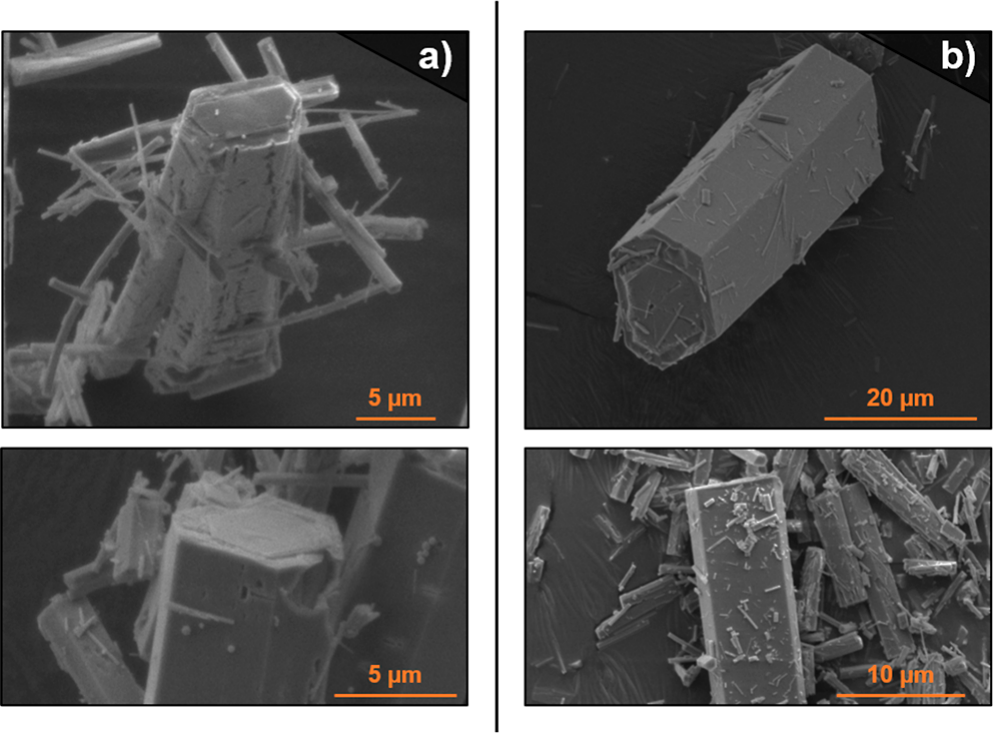
SEM images of (a) equimolar LaYb-RPF-4 synthesized in acetone/water and (b) equimolar LaGd-RPF-4 synthesized in acetone/water.

Furthermore, we also explored the possibility of modifying the synthetic medium as a way of altering the MOF formation kinetic factors and possibly leading to different metal distributions. Our new solvent of choice was acetone because the linker is highly soluble in it and due to its similar polarity to ethanol and miscibility with water. As the combinations of La with Yb and Gd in an ethanol/water mixture showed the most distinct features, we selected the same combinations for the acetone/water synthesis trials to verify whether the samples would behave in the same way in a slightly different synthetic medium. The results for the 9:1 and 1:9 binary combinations of lanthanum with ytterbium or gadolinium were congruent with the previous observations in a water/ethanol mixture. For La–Yb and La–Gd 9:1 initial ratios, the driving force that governs the distribution of the metals and the morphology of the crystals is the presence of lanthanum. The crystals displayed rough surfaces with defects and holes, and the average output was 9:1 in both cases. Correspondingly, synthetic reactions carried out with La–Yb and La–Gd 1:9 initial ratios showed the formation of crystals with defect-free morphologies similar to the single-metal Yb and Gd samples and with an average output composition of 1:9. However, for the equimolar initial ratios of both combinations, we found crystals that display a particular core–shell morphology in which the exterior layer contained a higher amount of lanthanum, while the inner core displayed an excess of the other metal. As for their aspect, the surface was not perfectly smooth as different ranges of defects could be observed on the bodies of the crystals, and these observations matched the lanthanum-rich exterior (Figure 9). This indicates that during the crystallization process, the initial nuclei that were formed were rich in the smaller-sized metal, in this case ytterbium and gadolinium, and the larger metal element, in this case lanthanum, was incorporated in a later stage in the surrounding growth. The compositional average output was found to be 7:3 for both combinations; therefore, there was an excess of lanthanum in the bulk of the samples. This suggests that when La-RPF-4 crystallizes over the already formed nuclei, it hinders its growth with another element, and also it inhibits any possible trans-metalation processes that could facilitate the insertion of other metals in the exterior layers.

**Figure 10:**
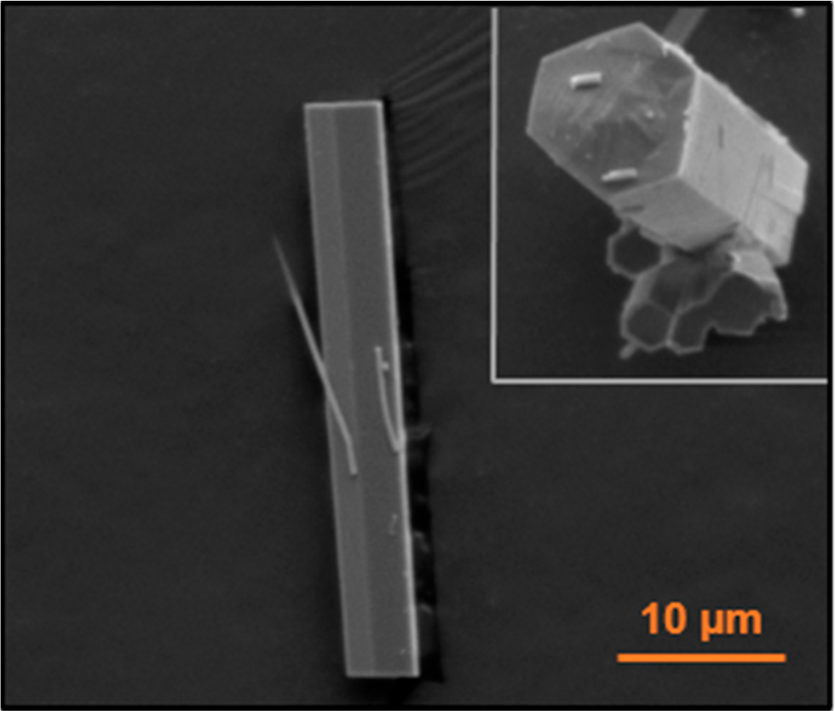
SEM images of the Yb–Er 1:1 (3 day) combination.

In order to get a more insightful view of the atomic distribution within the SBUs, NPD experiments were performed to obtain complementary information not accessible with X-ray diffraction due to the similar atomic number of the metal elements (Figures S13–S17). For these studies, we selected the combination of Er and Yb, as the contrast between their coherent scattering lengths (7.79 and 12.43 fm, respectively) allows one element to be easily distinguished from another. The experiments were performed at room temperature, using a vanadium sample carrier (broad peak at 70–74°). The Rietveld refinements were carried out using the Reflex module of the software Materials Studio 2019, and the starting atomic coordinates were provided by the single-metal reported structures. As expected, in the case of the samples with 1:9 and 9:1 combinations, the refinements converged when setting the chemical occupancy of the metal atoms to 0.1 and 0.9 for the corresponding elements. For the sample prepared from an equimolar ratio, other possible scenarios such as the formation of monometallic domains or cations alternated in the same rod that would bring about symmetry changes were ruled out, and a satisfactory fit was found for a 50% occupancy of each element at the same crystallographic site. The SEM–EDS analysis for this sample, synthesized in a reaction time of 3 days, showed no evidence of crystals with defects (Figure 10). Moreover, the EDS microanalysis revealed that the average output is in agreement with the initial combination, and this was also confirmed by the results of the TXRF bulk analysis (Table 1).

### Magnetic Characterization

Considering the structural features of the RPF-4 family comprising rod-shaped SBUs made of lanthanide ions at short distances (from 3.6 to 3.9 Å), the magnetic properties of the whole series of compounds were also evaluated to investigate any possible long-range magnetic interaction between the lanthanide cations as well as differences in the magnetic behavior between single-metal and multi-metal MTV-MOFs.

**Figure 11:**
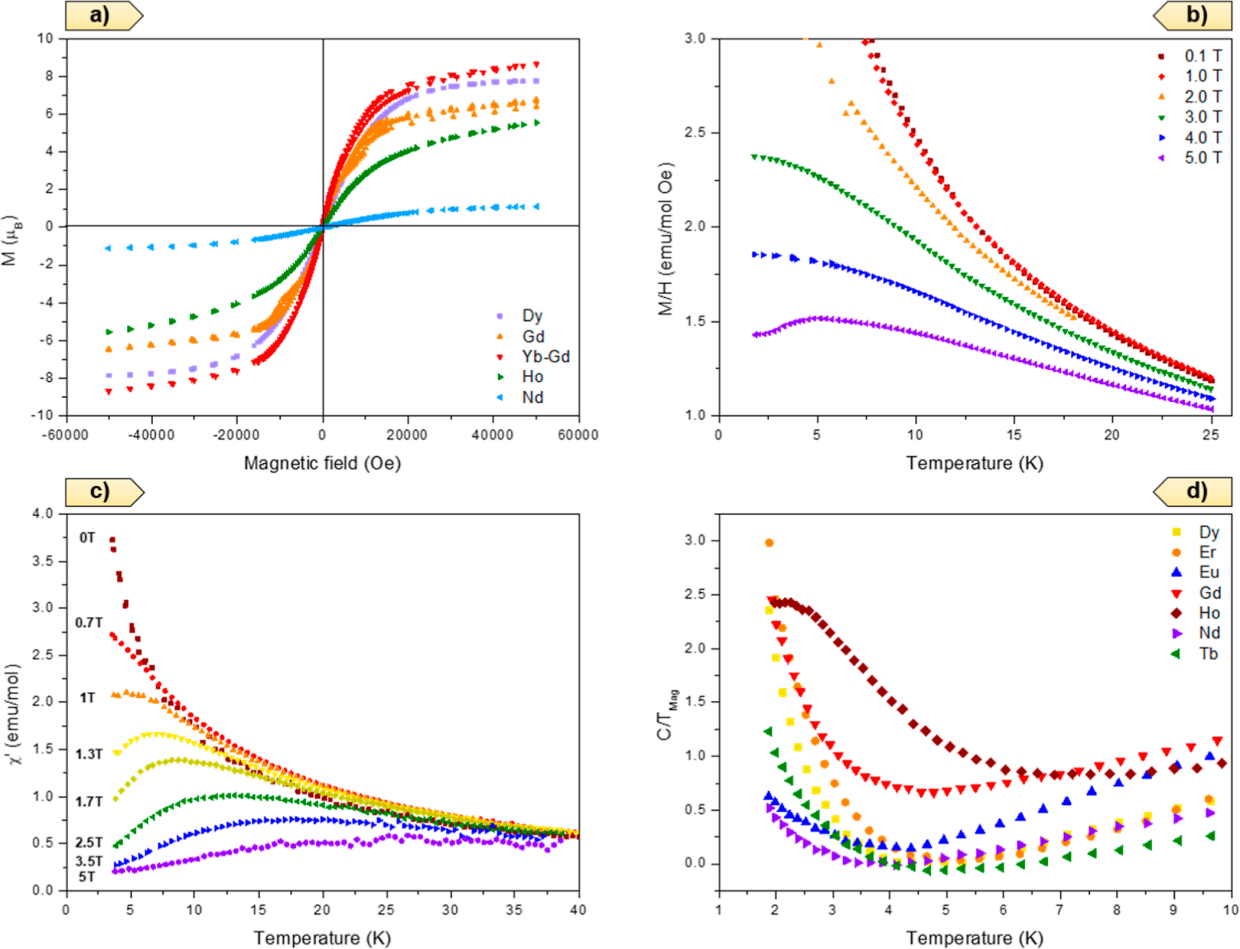
(a) Magnetic Field dependence of the magnetization at 1.8 K for different compounds in the series; (b) temperature dependence of the magnetic susceptibility at different measuring magnetic fields in the range of 0.1 to 5 T for Gd-RPF-4; (c) temperature dependence of the AC magnetic susceptibility at a fixed frequency of 10 KHz and amplitude of 1 Oe for different external magnetic fields for Gd-RPF-4; (d) temperature dependence, in the log scale, of the magnetic component of the specific heat for different members of the RPF-4 family.

The magnetic susceptibility, measured with a moderate applied field of 100 Oe, presents a paramagnetic behavior in the range 1.8 to 300 K. Also, from the inverse magnetic susceptibility, we were able to fit to a Curie Weiss linear law behavior in many cases and calculate the paramagnetic moment on the rare-earth atoms as well as the Curie constant (Figure S19 for the magnetic susceptibility graph and Table 2). In most of the cases, the measured paramagnetic moment per rare-earth atom was close to the expected value, except in the case of Eu-RPF-4, probably due to the well-known different magnetic states of this particular ion. Some complementary data on the temperature dependence of the inverse magnetic susceptibility for different members of the series is also shown in Figure S19. In addition, the magnetic field dependence at a fixed temperature (1.8 K) of the magnetization (Figure 11a) indicate a saturated moment, which depends on the rare-earth ion. No member of the series presented a coercive field in the M versus H curve. This behavior was probably related to the polarization effect on the paramagnetic rare-earth atom under the intense external applied magnetic field.

**Table 2:** Curie Constant (K), Paramagnetic Moment (μ_B_) per Formula Unit or per Rare-Earth Atom, Saturation Magnetization (μ_B_) at 1.8 K per Atom, and Expected (Theoretical) Effective Paramagnetic Moment (μ_B_) per Rare-Earth Atom for the Different Series of Rare-Earth Ions and Binary Combinations^a^

	Θ (K)	PM (μ_B_/f.u.)	PM (μ_B_/atom)	*M*_S_ (μ_B_/atom) @1.8 K	PM (μ_B_)_Efective_	*T*_Max_ (K) from AC
Ce	–5.0	2.8	2.0	0.9	2.5	0.4
Nd	–58.0	5.3	3.7	1.2	3.6	0.2
Eu	–192.0	4.8	3.4	0	0	1.2
Gd	–9.0	15.8	11.0	7.0	7.9	0.5
Tb	+15.0	13.0	9.2	6.0	9.8	3.7
Dy	+0.5	11.3	8.0	8.0	10.5	0.6
Ho	–22.4	16.7	11.8	6.0	10.6	0.6
Er	–21.0	14.8	10.4	8.0	9.6	1.6
Yb	–191.0	7.4	5.2	1.9	4.5	0.4
Yb–Gd	–9.4	9.8		10.0^b^	9.1	2.7
Yb–Dy	–4.9	11.5		8.0^b^	11.4	1.6

a Also includes the extrapolated T_Max_ from the AC magnetic susceptibility data.

b *M*_S_ (μ_B_/f.u.).

The sign of the magnetic interactions extracted from the Curie Constant indicate not very strong values, mostly antiferromagnetic (negative values). However, in two cases (Tb and Dy), the data present rather weak ferromagnetic-like interactions. We also measured the DC magnetic susceptibility under very different applied magnetic fields in the range from 0 to 5 T (Figure 11b), in the temperature range from 2 to 25 K. For the low magnetic field, we could suggest that a maximum was present at a very low temperature (below 2 K), shifting to a higher temperature when the applied magnetic field increased, with a broad cusp-like maximum clearly visible above 2 K. This behavior is not expected for a simple paramagnetic ion under different magnetic fields and is probably related to a certain type of magnetic interaction between the low-lying energy levels of the lanthanides, which could be split by the crystal and the magnetic field.

In order to detangle the intrinsic magnetic behavior, we performed measurements of the AC magnetic susceptibility with a magnetic field amplitude of 1 Oe under a wide range of frequencies (from 10 Hz to 10 kHz), with the possibility to superimpose a significant external DC magnetic field. The AC magnetic susceptibility measurements, with only the sinusoidal magnetic field, showed a clear paramagnetic behavior. However, a maximum (broad cusp-like) was also observed (Figure 11c) at a given temperature (*T*_Max_), which shifted to higher values when a significant external magnetic field was applied. This peak was clearly observed for external DC magnetic fields higher than 0.5 T, up to almost 5 T. The field dependence of this temperature maximum (*T*_Max_) followed a linear behavior (Figure S20), from which we extrapolated a *T*_Max_ for a zero magnetic field as 0.53 K (in the case of Gd-RPF4) as well as a slope in *T*_Max_ in the order of 4.7 K/Tesla. The summary of the *T*_Max_ at a zero magnetic field in each case is presented in Table 2; the value of *T*_Max_ (0 T) was always below our experimental accessible temperature range (above 2 K) except in the cases of Tb-RPF-4 and Yb-Gd-RPF-4.

**Figure 12:**
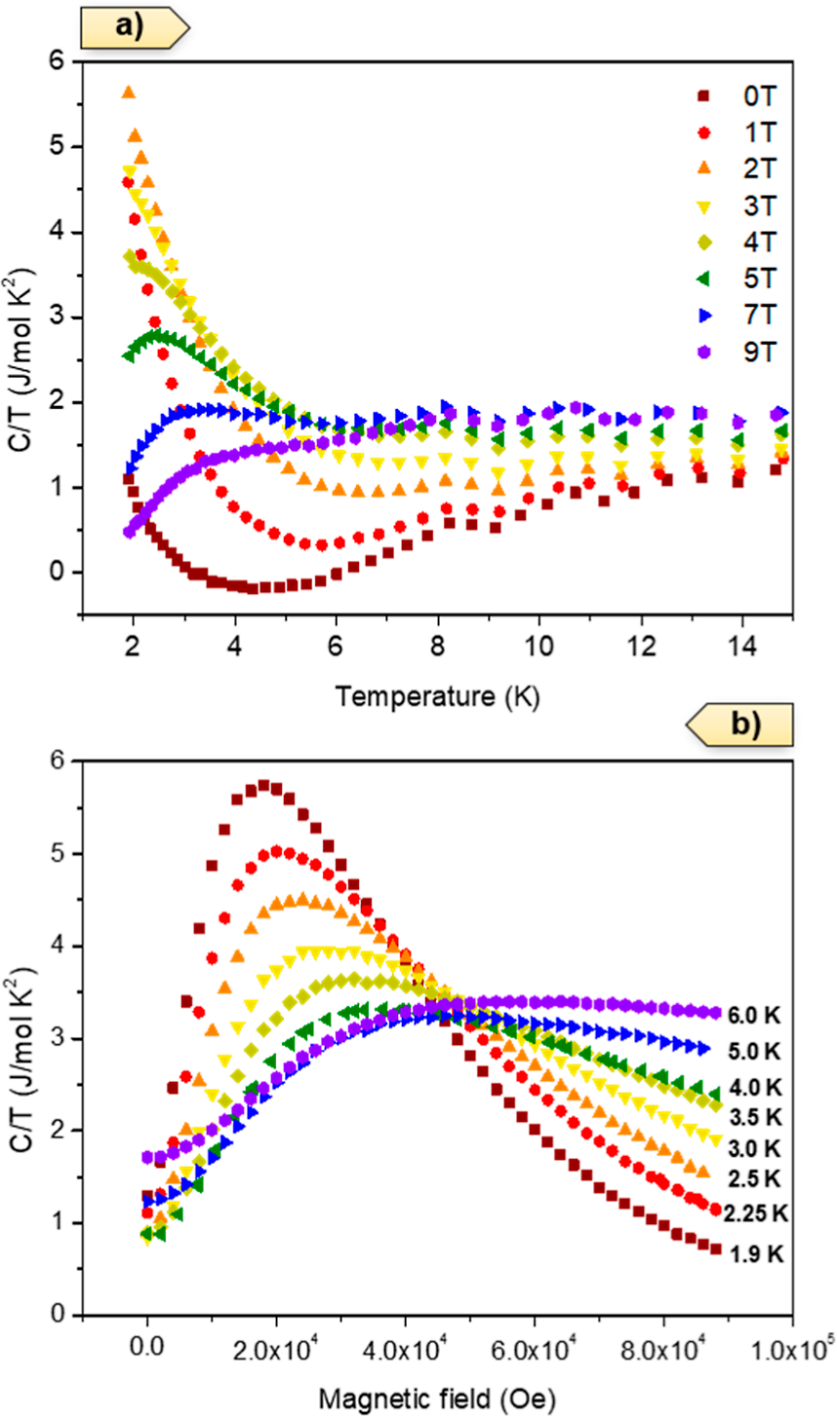
(a) Temperature dependence of the magnetic specific heat of the Yb/Gd-RPF-4 sample under different magnetic fields and (b) magnetic field dependence of the magnetic component of the specific heat at different temperatures (isothermal).

In summary, the magnetic susceptibility (AC and DC) under different external magnetic fields seemed to indicate a complex magnetic behavior related to the interaction between the lower energy crystal field levels associated with the lanthanide ions. In order to clarify this point, we measured in detail the specific heat (*C*_p_) of the different members of the series because the low temperature-specific heat is very sensitive to the transitions between the low energy crystal field levels in lanthanides (Schottky anomalies). In principle, at low temperatures, the specific heat is composed of three components: lattice vibrations (phonons), electronic component (extremely weak in insulators), and the magnetic contribution. To extract the magnetic component of the specific heat, from the specific heat of each compound, we subtracted the heat capacity obtained from the La-RPF-4 sample, which only presented the phonon contribution to the specific heat but not the magnetic component related to the magnetic moment and the crystal field level transition related to the magnetic rare-earth ions. In all cases, we assumed that the electronic component of the specific heat was extremely weak due to the insulating character of the compounds. The magnetic component of the specific heat versus temperature in the range below 20 K is shown in Figure 11d. Depending on the rare-earth ions, the value of the magnetic component of C/T strongly increased below 3 K, and in the case of Ho-RPF-4, it showed a broad maximum at around 2.3 K. In order to shed some light into this complex behavior, we studied the magnetic field dependence of the specific heat in the same temperature range. For instance, in the case of Yb-Gd-RPF-4, we measured the specific heat under different applied external magnetic fields in the range from 0 to 9 T (Figure 12a). The data showed clearly that at magnetic fields higher than 3 T, a broad maximum in the magnetic specific heat was observed in the measured temperature range. Also, for each C/T data at a given applied magnetic field, we could subtract the C/T the data from *H* = 0 T in order to emphasize the real effect of the applied magnetic field. The data are presented in Figure S21. The observed broad maximum in C/T under the applied magnetic field was much more pronounced, but not very different information was obtained. As a complementary approach, we measured the isothermal magnetic field dependence of the magnetic specific heat (Figure 12b). A maximum in the specific heat was observed below 4 T, for temperatures below 5 K, with the *H*_Max_ shifted to a higher field as the temperature increased from 1.9 to 5 K.

From these data, we could extract the magnetic field dependence of T_Max_, showing a linear behavior that indicated a maximum at approximately *T*_Max_ = 0.4 K at a zero magnetic field (see Figure S22). In principle, this behavior is consistent with the magnetic susceptibility data, but at slightly different temperatures, again pointing to transitions between low crystal field energy levels at the rare-earth ions. The integration of these signals (*C*/*T*)_Mag_ is related to magnetic entropy, which depends on the different rare-earth ions, as presented in the Supporting Information in Figure S23. The magnetic component of the specific heat under applied external magnetic fields showed a very broad maximum, which in principle was not related to long-range magnetic order. However, in order to definitely rule out this possibility, we performed a neutron diffraction experiment at a very low temperature (well below 1.5 K). Clearly, the NPD measurement collected at 84 mK with the Er-RPF-4 sample (as an example) neither showed any extra diffraction peaks nor an unusual increase of intensity of same diffraction peaks, which we could associate with a magnetic phase transition. Therefore, the magnetic field-dependent specific heat broad maximum must have been arising from a very complex temperature dependence of the rare-earth crystal field-level transitions, depending on the different rare-earth ions. In general, rare-earth-containing compounds often display such a low temperature peak in specific heat, which can be due to the few-level electronic system of the partially split ground-state manifold of the rare-earth 4f-electrons.^51^ These levels are modified by both the applied magnetic field and the crystal field of the host. There is a low temperature characteristic peak, with a very strong magnetic field dependence, in the specific heat for the complete series of lanthanide compounds either with a single rare-earth cation or in some cases even with a mixture of rare-earth ions. The low-temperature, magnetic field-dependent, specific heat feature was analyzed in terms of the Schottky-model of a two-level system.^52^ First, the effect of the host was removed to a certain extent. The heat capacity of the matrix (phonons) is typically accounted for by measuring a non-magnetic rare-earth-filled variant, typically with La or Lu.^53^ Here, La-RPF-4 was measured as a background for the mass normalized specific heat. Indeed, it did not show any low-temperature anomaly or magnetic field dependence of the heat capacity.^54^ The Supporting Information gives an illustrative example, using the excess magnetic specific heat in various magnetic fields of Er-RPF-4, in Figure S24 of these two-level analyses. Figures S24–S28 show the estimated magnetic field-dependent two-level energy gaps, Δ*E*_g_(B), grouped according to the detailed Schottky-model used. Table 3 gathers the estimated two-level energy gap without a magnetic field, Δ*E*_g_, and its magnetic field dependence for the lanthanide series of single-metal, and a few selected multi-metal RPF-4 compounds. Since the estimated gap does not always vary linearly with the magnetic field, Table 3 indicates *E*_g_*B*[K/T] = (Δ*E*_g_(*B*) – Δ*E*_g_)/*B* using the heat capacity measured in the highest measured field (usually 8.5 T). Table 3 is ordered according to the zero-field gap and can be compared to the 4f configuration. Interestingly, the multi-metal YbDy-RPF-4 and YbGd-RPF-4 compounds had similar gaps as the single-metal Yb-RPF-4 even though both Gd-RPF-4 and Dy-RPF-4 showed considerably larger gaps.

**Table 3:** Estimated Two-Level Schottky-Model Energy Gap without a Magnetic Field (Δ*E*_g_) in Temperature Units and Its Magnetic Field Dependence (*E*_g_*B*) for Single and Multi-Metal RPF-4

RPF-4	Δ*E*_g_[K]	*E*_g_*B*[K/T]
La-RPF-4	0	0
Nd-RPF-4	0.73	0.9
Ce-RPF-4	1.6	0.8
Yb-RPF-4	1.9	1.3
YbDy-RPF-4	2.1	0.8
YbGd-RPF-4	2.5	0.9
Er-RPF-4	3.4	1.1
Gd-RPF-4	3.6	1.2
Dy-RPF-4	4.9	0.7
Tb-RPF-4	5.2	0.16
Eu-RPF-4	5.9	0.21
Ho-RPF-4	9.1	0.9

In summary, the results of this work illustrate how the synthesis and crystallization processes of multi-metal MTV-MOFs are determinant for the incorporation of the different metal elements into the MOF building units. While compositional analysis of a bulk sample might indicate a homogeneous distribution of the metal elements, a careful analysis of single crystal compositions reveals that a compositional segregation in MOF crystals appears, even for combinations of elements for which the same MOF can be obtained in the single-metal form, driven by the complex interplay between thermodynamic and kinetic crystallization factors. Besides the relative short distance between rare-earth cations, for instance, similar to distances between 3d cations in different typical inorganic oxides, the MTV-RPF-4 stays in the paramagnetic regime until the lowest temperature. However, the interaction between the low-lying levels of the f-electron strongly depends on the variations induced by the crystal field and the external applied magnetic field, giving rise to a rich phenomenology in the magnetic susceptibility (AC and DC) and also in the specific heat.

## Supporting Information Available

Additional experimental details, including PXRD patterns, NPD refinement graphs, EDS spectra, magnetic measurements details and graphs, and computational details (PDF).
