# Supplementary material for: Influence of the Synthesis and Crystallization Processes on the Cation Distribution in a Series of Multivariate Rare-Earth Metal–Organic Frameworks and Their Magnetic Characterization
Source: Chem Mater. 2022 Jul 25;34(15):7029–41. doi: 10.1021/acs.chemmater.2c01481 (PMC9367679; doi:10.1021/acs.chemmater.2c01481)
Supplement: Supplementary file 1 — cm2c01481_si_001.pdf [file cm2c01481_si_001.pdf]

Supporting Information

Influence of the Synthesis and Crystallization Processes on the Cation Distribution in a Series of Multivariate Rare-earth Metal-organic Frameworks, and their Magnetic Characterization.

Raluca Loredana Vasile,[†] *Agustín Alejandro Godoy,*[‡] *Inés Puente Orench,*[§] *Norbert M. Nemes,*[ø] *Víctor A. de la Peña O'Shea,*[þ] *Enrique Gutiérrez-Puebla,*[†] *Jose Luis Martínez*[†] *M. Ángeles Monge*[†*] *Felipe Gándara*[†*]

† Materials Science Institute of Madrid – Spanish National Research Council (ICMM-CSIC). Calle Sor Juana Inés de la Cruz 3, 28049, Madrid, Spain.

‡ Instituto de Investigaciones en Tecnología Química (INTEQUI-CONICET), Universidad Nacional de San Luis, Alte. Brown 1450, D5700HGC San Luis, Argentina.

§ Institut Laue Langevin, 71 Avenue des Martyrs, Grenoble, 38042, France and Instituto de Nanociencia y Materiales de Aragón (INMA-CSIC), Calle Pedro Cerbuna 12 50009 Zaragoza, Spain.

ø Departamento de Física de Materiales, Facultad Físicas. Universidad Complutense de Madrid, E-28040 Madrid, Spain.

þ Photoactivated Processes Unit IMDEA Energy Institute, Móstoles Technology Park, Avenida Ramón de la Sagra 3, Móstoles, Madrid, 28935, Spain.

INDEX

Powder X-ray Diffraction (PXRD) **1**

Energy Dispersive X-ray Spectroscopy (EDS) **8**

Neutron Powder Diffraction (NPD) **10**

Synthetic details **13**

Magnetic characterization **14**

Computational details **23**

Powder X-ray Diffraction (PXRD)

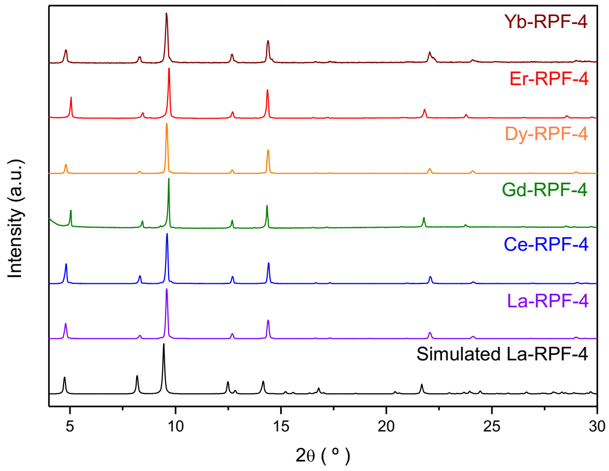

Figure S1. Powder X-ray diffraction patterns of the single-metal RPF-4 samples.

a)

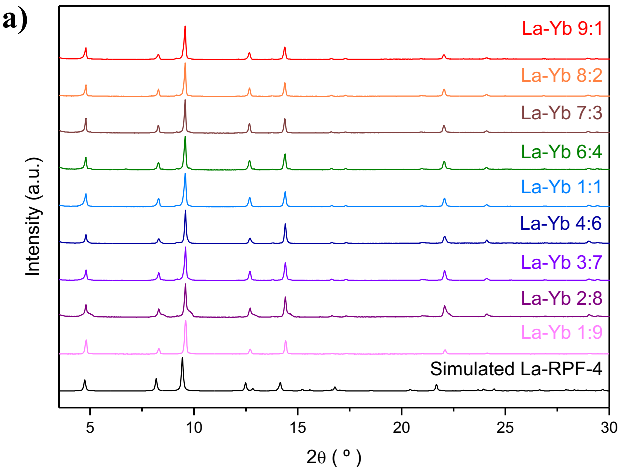

b)

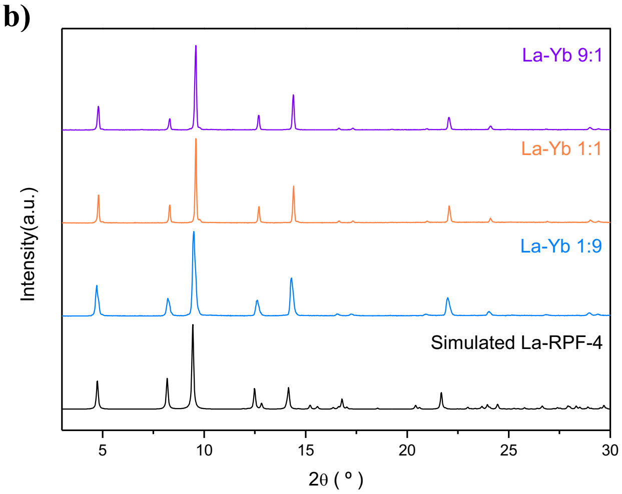

Figure S2. LaYb-RPF-4 powder X-ray diffraction patterns: **a)** samples synthesized in a reaction time of 1 day **b)** samples synthesized in a reaction time of 3 days.

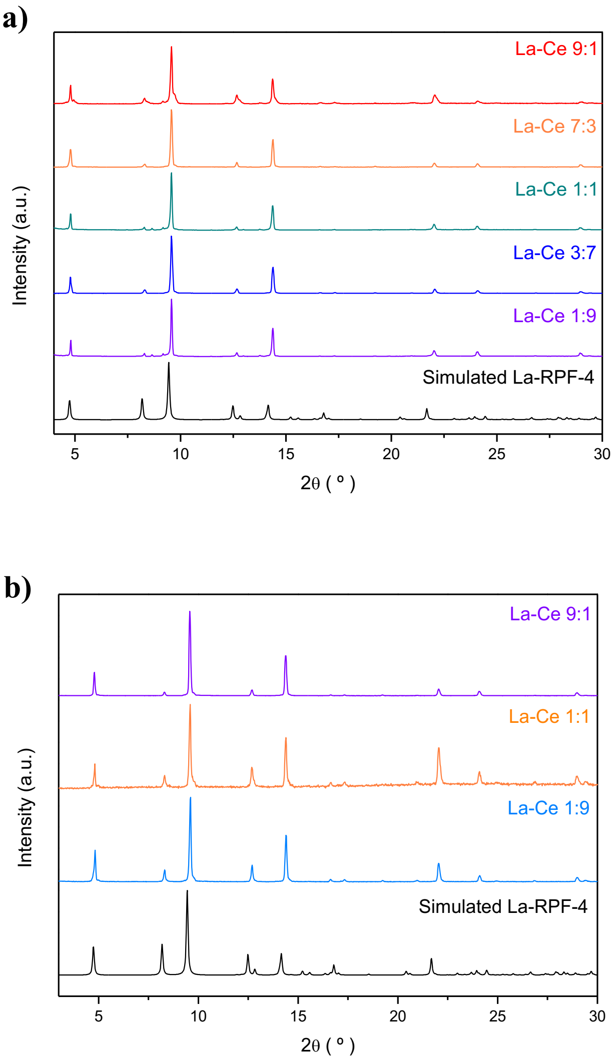

Figure S3. LaCe-RPF-4 powder X-ray diffraction patterns: **a)** samples synthesized in a reaction time of 1 day **b)** samples synthesized in a reaction time of 3 days.

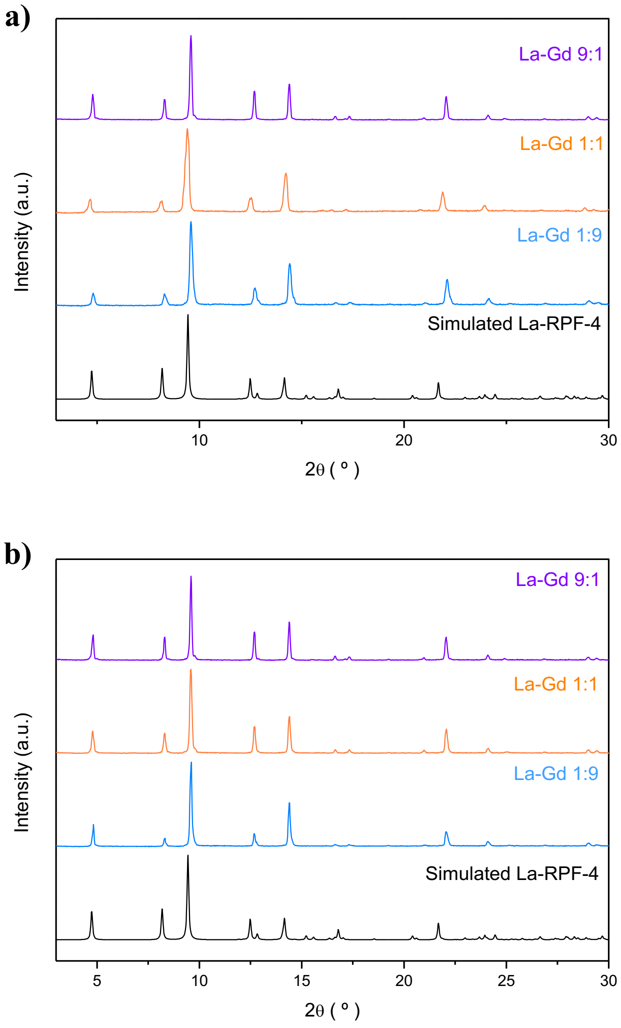

Figure S4. LaGd-RPF-4 powder X-ray diffraction patterns: **a)** samples synthesized in a reaction time of 1 day **b)** samples synthesized in a reaction time of 3 days.

a)

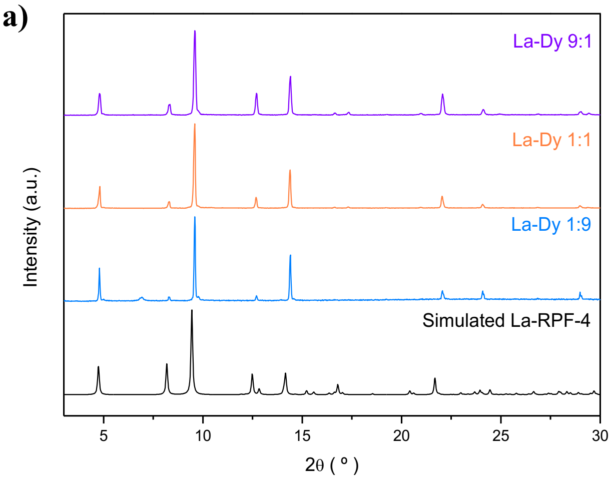

b)

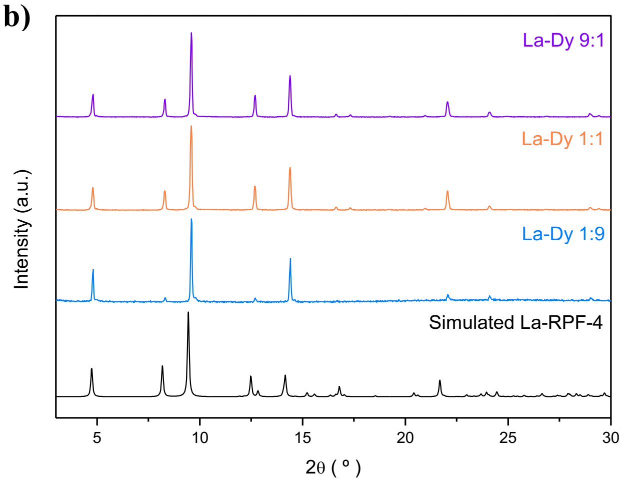

Figure S5. LaDy-RPF-4 powder X-ray diffraction patterns: **a)** samples synthesized in a reaction time of 1 day **b)** samples synthesized in a reaction time of 3 days.

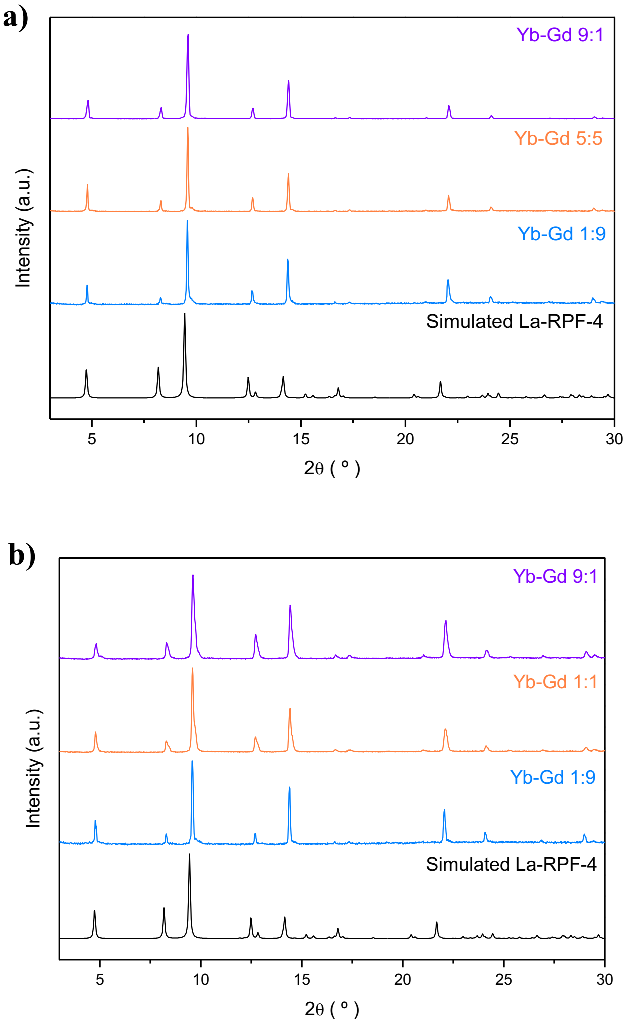

Figure S6. YbGd-RPF-4 powder X-ray diffraction patterns: **a)** samples synthesized in a reaction time of 1 day **b)** samples synthesized in a reaction time of 3 days.

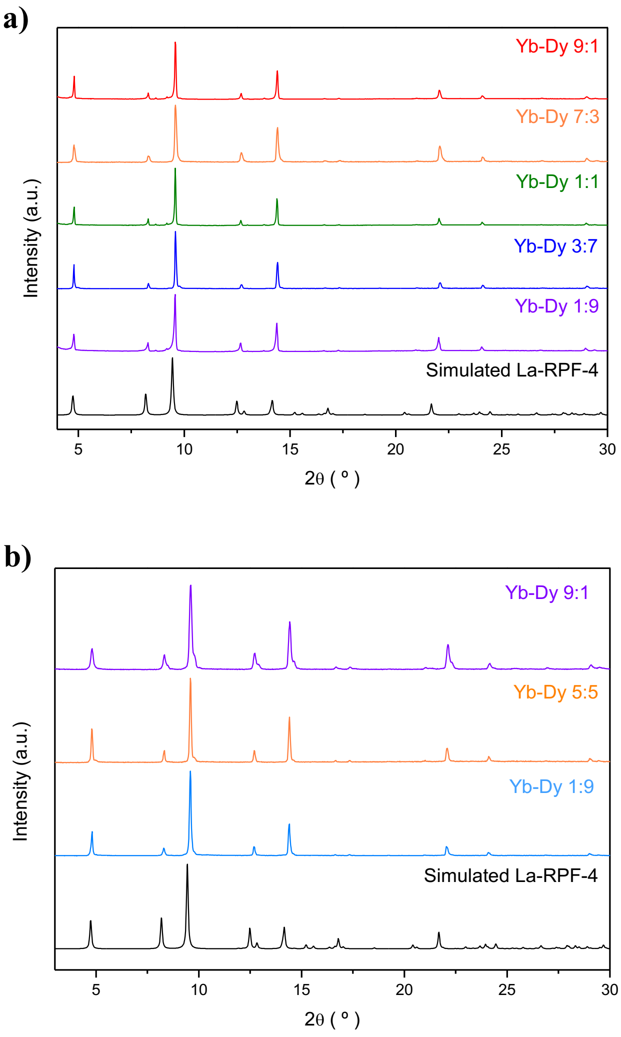

Figure S7. YbDy-RPF-4 powder X-ray diffraction patterns: **a)** samples synthesized in a reaction time of 1 day **b)** samples synthesized in a reaction time of 3 days.

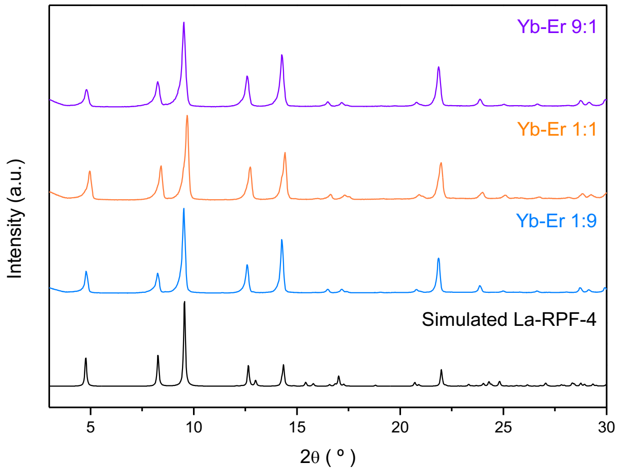

Figure S8. YbEr-RPF-4 powder X-ray diffraction patterns, samples synthesized in a reaction time of 3 days.

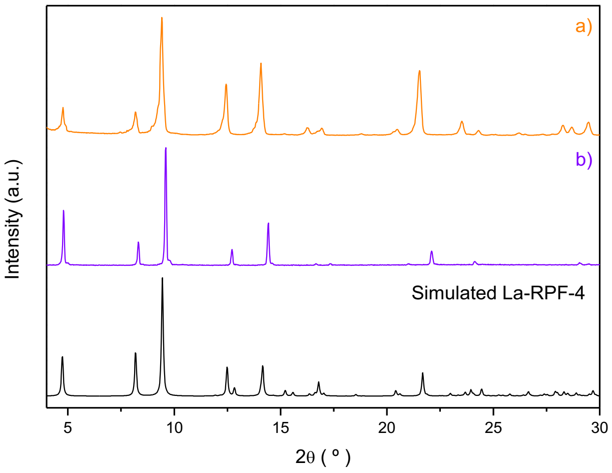

Figure S9. Powder X-ray diffraction patterns of the seeding experiments: **a)** La-RPF-4 + $Yb(NO_3)_3$ x5H_2O **b)** Yb-RPF-4 + $La(NO_3)_3$ x6H_2O

a)

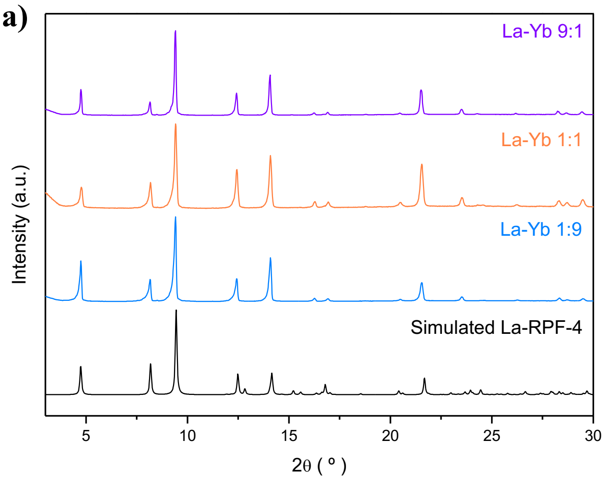

b)

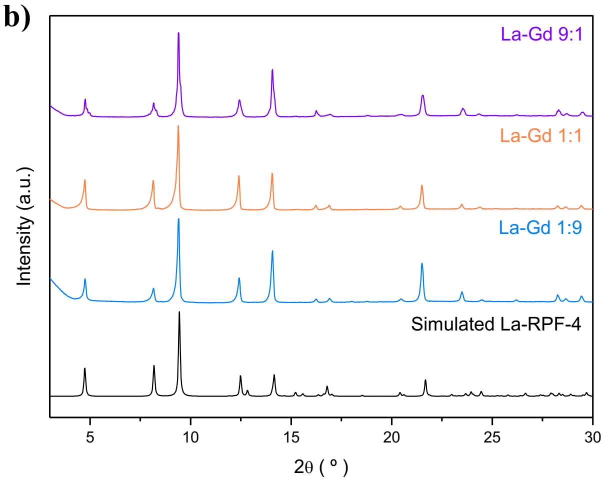

Figure S10. Different solvent mixture experiments synthesized in a reaction time of 1 day **a)** LaYb-RPF-4 and **b)** LaGd-RPF-4.

Energy Dispersive X-ray Spectroscopy (EDS)

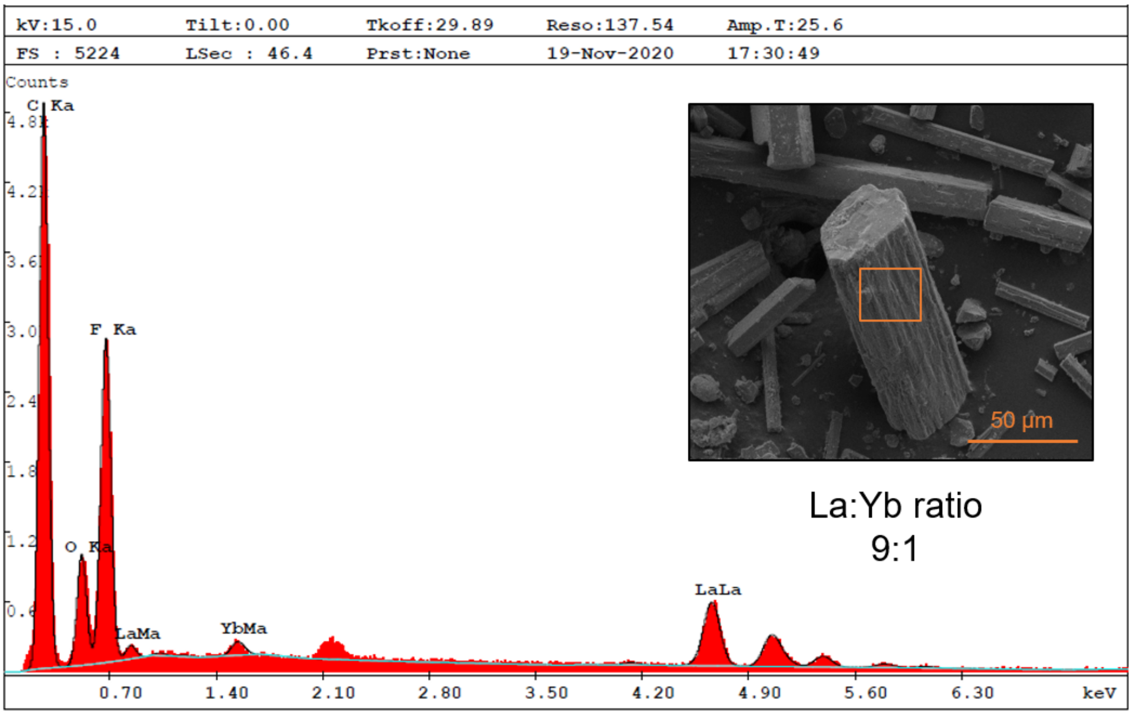

Figure S11. EDS spectrum of the seeding experiment: La-RPF-4 + $Yb(NO_3)_3$ x$5H_2O$.

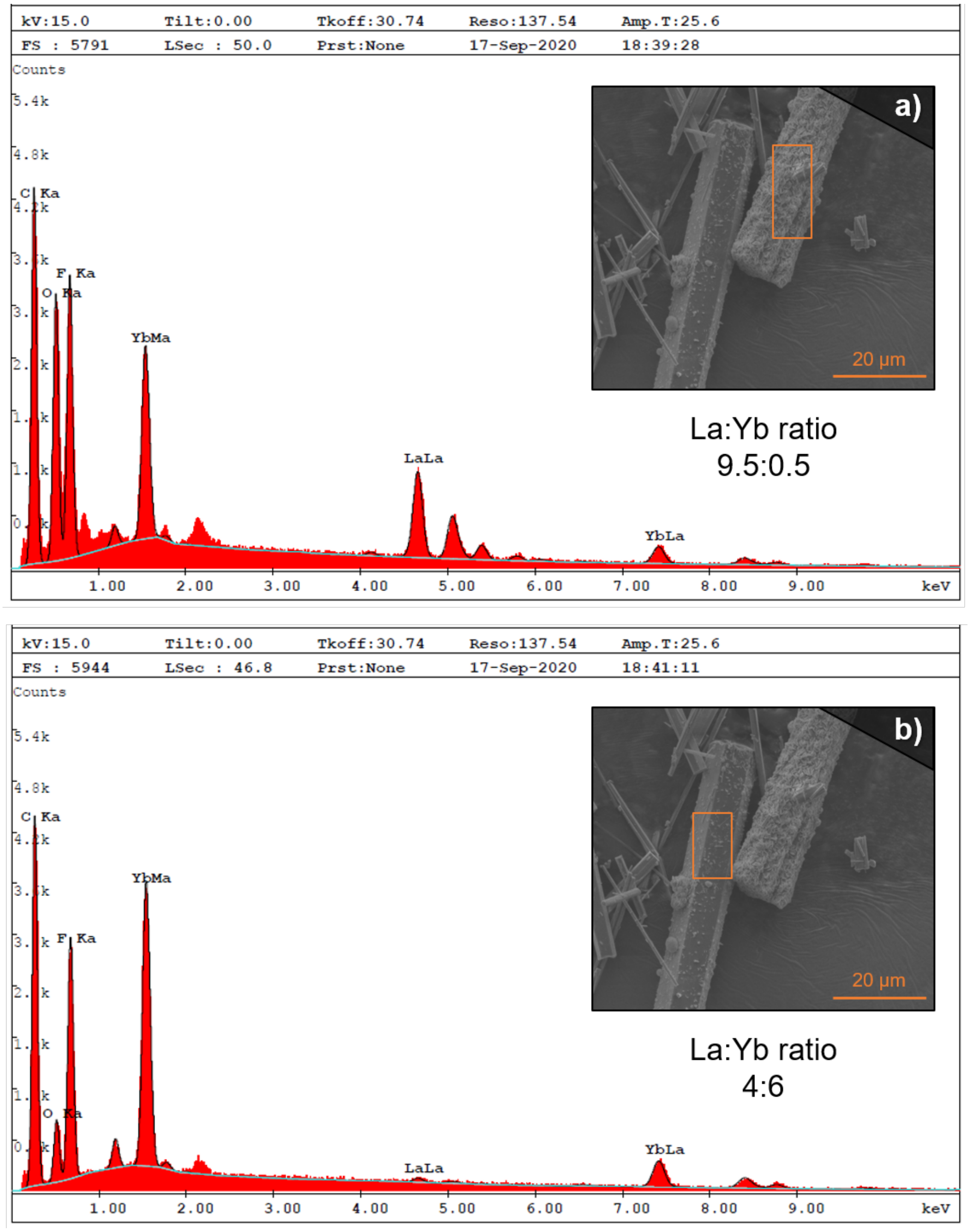

Figure S12. EDS spectrum of the seeding experiment: Yb-RPF-4 + $La(NO_3)_3$ x6H_2O.

Neutron Powder Diffraction (NPD)

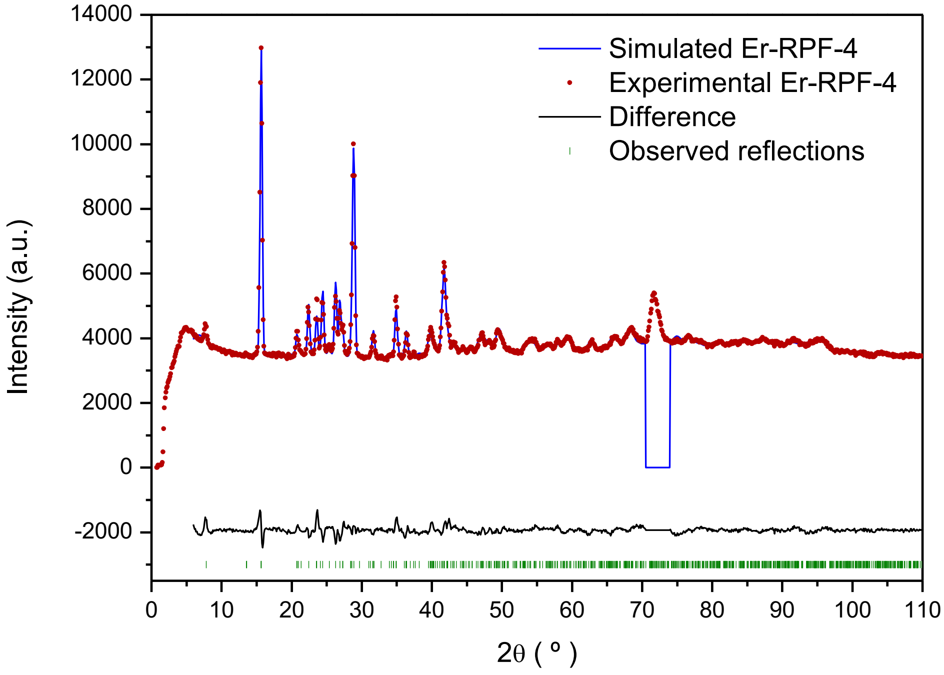

Figure S13. Neutron powder diffraction (NPD) pattern and Rietveld refinement of Er-RPF-4. Rwp = 2.23%; Rp = 1.57%.

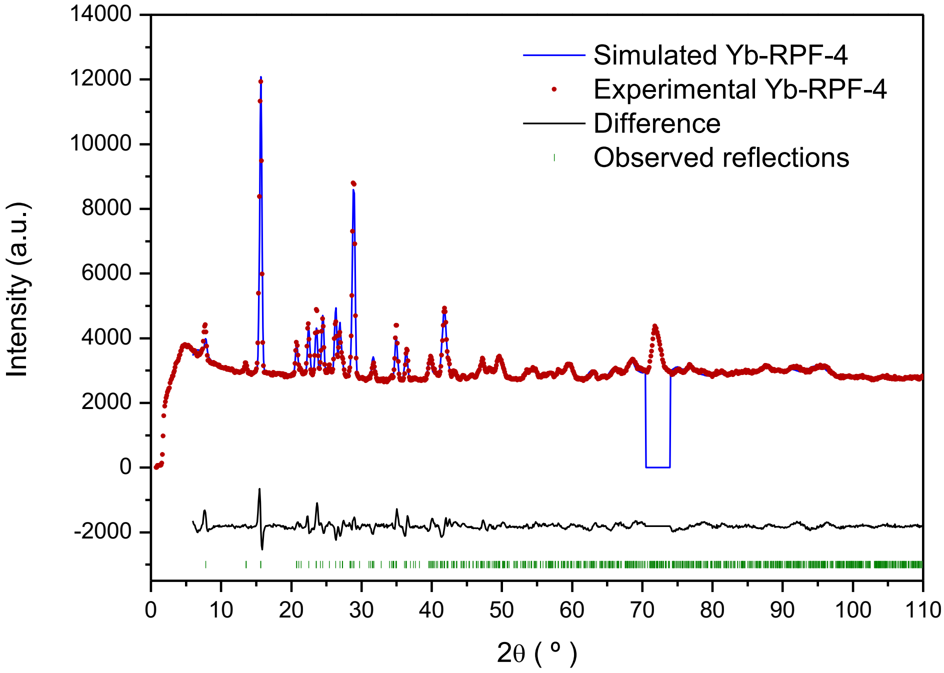

Figure S14. Neutron powder diffraction (NPD) pattern and Rietveld refinement of Yb-RPF-4. Rwp = 3.10%; Rp = 2.10%.

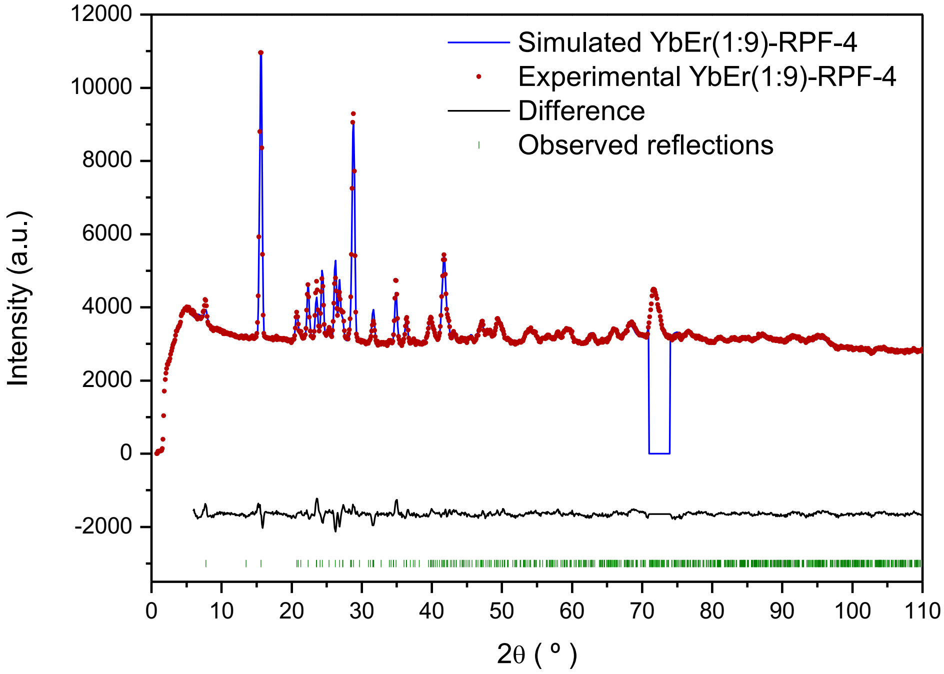

Figure S15. Neutron powder diffraction (NPD) pattern and Rietveld refinement of YbEr(1:9)-RPF-4. Rwp = 2.06%; Rp = 1.46%.

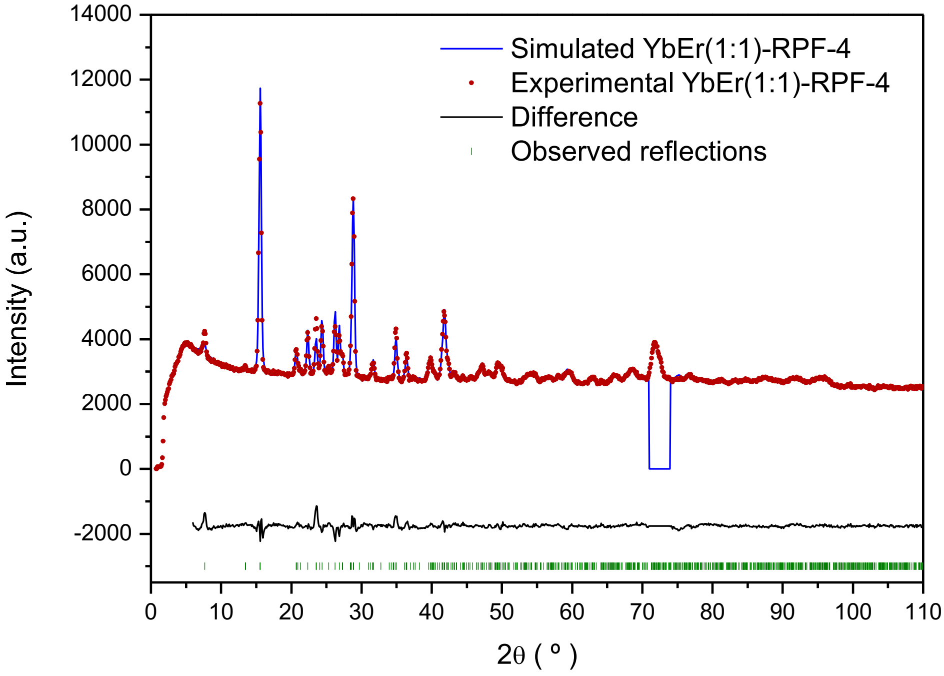

Figure S16. Neutron powder diffraction (NPD) pattern and Rietveld refinement of YbEr(1:1)-RPF-4. Rwp = 2.15%; Rp = 1.45%.

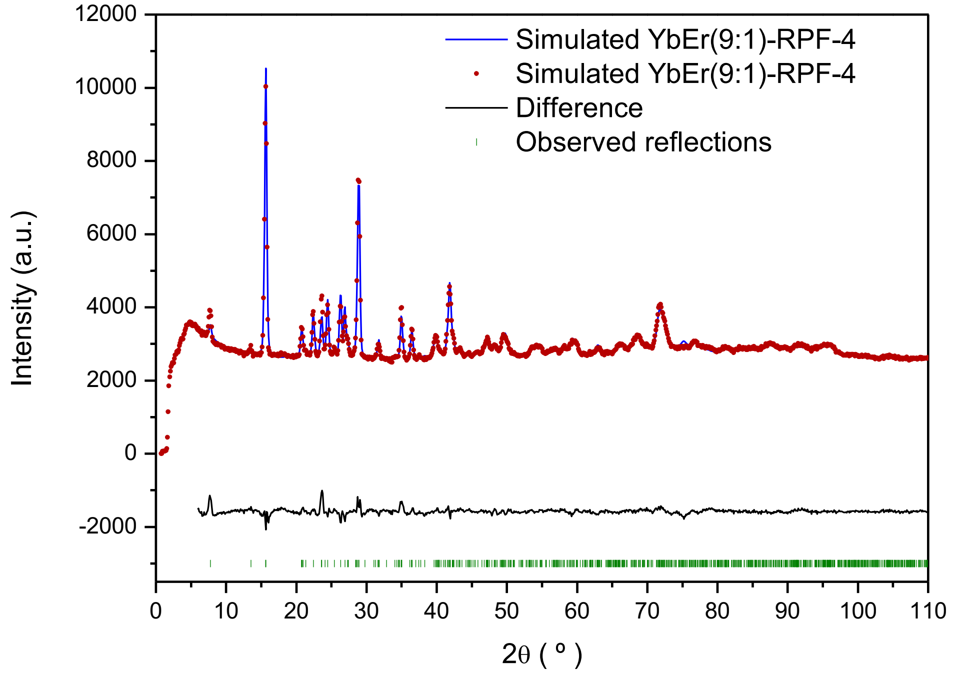

Figure S17. Neutron powder diffraction (NPD) pattern and Rietveld refinement of YbEr(9:1)-RPF-4. Rwp = 2.13%; Rp = 1.44%.

Synthetic details

Table S1. Synthesis ratios for the RPF-4 combinations.

La-Yb	Molar code	$La(NO_3)_3$ x $6H_2O$		$Yb(NO_3)_3$ x $5H_2O$	
		mg	mmol	mg	mmol
	1:9	4.98	0.01	46.53	0.1
	2:8	9.97	0.02	41.36	0.09
	3:7	14.95	0.03	36.19	0.08
	4:6	19.94	0.05	31.02	0.07
	1:1	24.92	0.06	25.85	0.06
	6:4	29.91	0.07	20.68	0.05
	7:3	34.89	0.08	15.51	0.03
	8:2	39.88	0.09	10.34	0.02
	9:1	44.86	0.1	5.17	0.01

La-Ce	Molar code	$La(NO_3)_3$ x $6H_2O$		$Ce(NO_3)_3$ x $6H_2O$	
		mg	mmol	mg	mmol
	1:9	4.98	0.01	45.39	0.1
	3:7	14.95	0.03	35.30	0.08
	1:1	24.92	0.06	25.22	0.06
	7:3	34.89	0.08	15.13	0.03
	9:1	44.86	0.1	5.04	0.01

La-Gd	Molar code	$La(NO_3)_3$ x $6H_2O$		$Gd(NO_3)_3$ x $6H_2O$	
		mg	mmol	mg	mmol
	1:9	4.98	0.01	46.77	0.1
	1:1	24.92	0.06	25.98	0.06
	9:1	44.86	0.1	5.20	0.01

La-Dy	Molar code	$La(NO_3)_3$ x $6H_2O$		$Dy(NO_3)_3$ x $6H_2O$	
		mg	mmol	mg	mmol
	1:9	4.98	0.01	47.31	0.1
	1:1	24.92	0.06	26.28	0.06
	9:1	44.86	0.1	5.26	0.01

Yb-Gd	Molar code	$Yb(NO_3)_3$ x $5H_2O$		$Gd(NO_3)_3$ x $6H_2O$	
		mg	mmol	mg	mmol
	1:9	5.17	0.1	46.77	0.01
	1:1	25.85	0.06	25.98	0.06
	9:1	46.53	0.01	5.20	0.1

Yb-Dy	Molar code	$Yb(NO_3)_3$ x $5H_2O$		$Dy(NO_3)_3$ x $6H_2O$	
		mg	mmol	mg	mmol
	1:9	5.17	0.1	47.31	0.1
	1:1	25.85	0.06	26.28	0.06
	9:1	46.53	0.01	5.26	0.01

Yb-Er	Molar code	$Yb(NO_3)_3$ x $5H_2O$		$Er(NO_3)_3$ x $5H_2O$	
		mg	mmol	mg	mmol
	1:1	25.85	0.06	25.49	0.06

Magnetic characterization

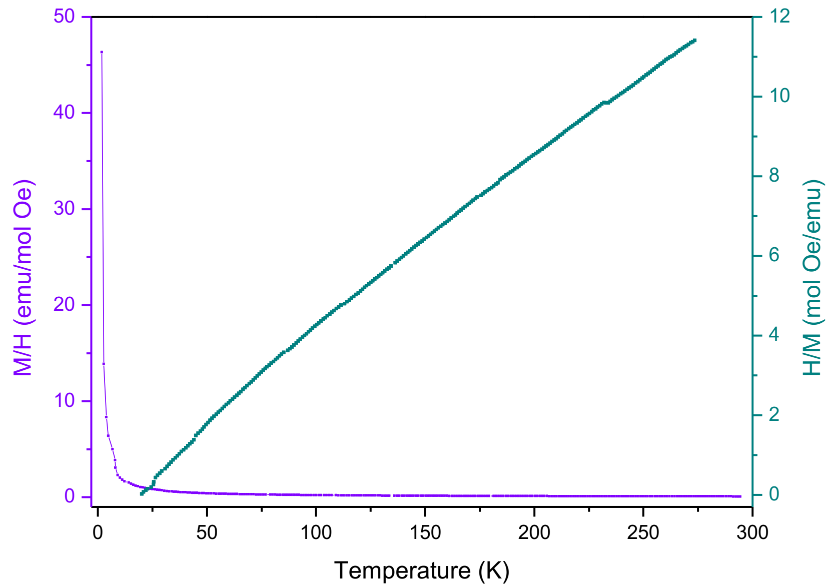

Figure S18. Temperature dependence of the DC magnetic susceptibility for Er-RPF-4.

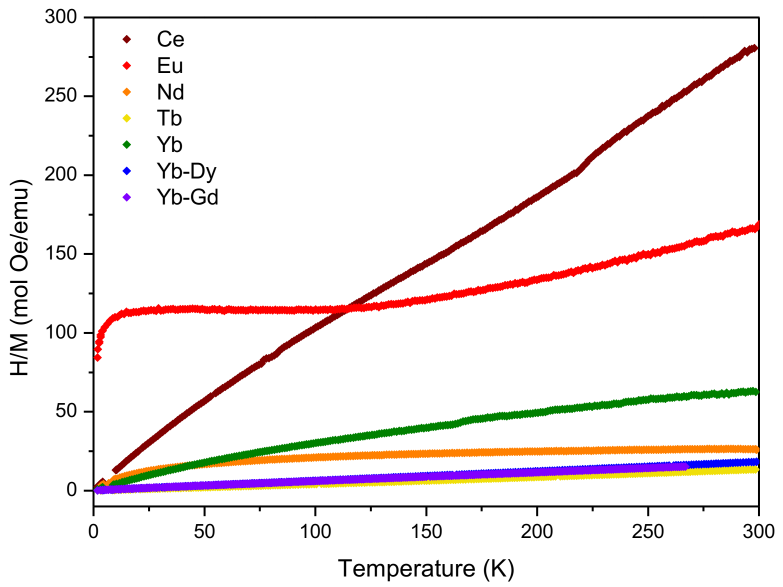

Figure S19. Temperature dependence on the Inverse Magnetic Susceptibility for different compounds of the RPF-4 series.

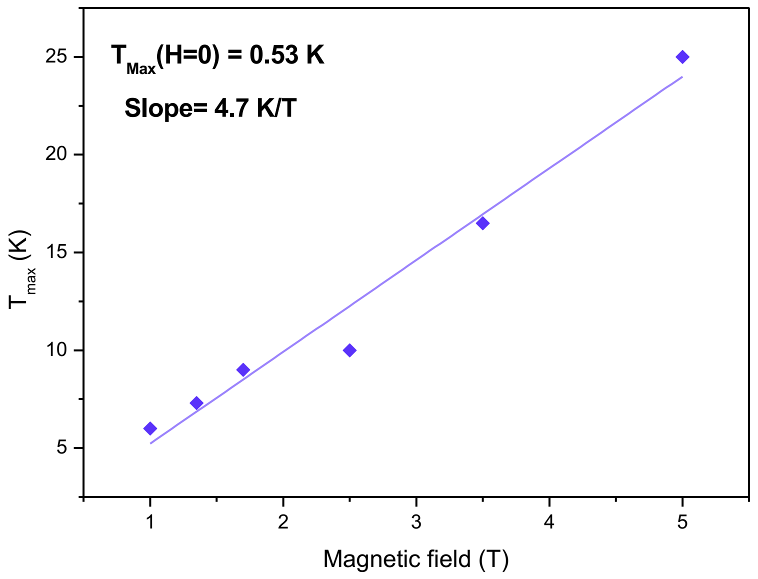

Figure S20. Magnetic field dependence of the temperature of the maximum in AC magnetic susceptibility for Gd-RPF-4. The straight line is a linear fitting of the data.

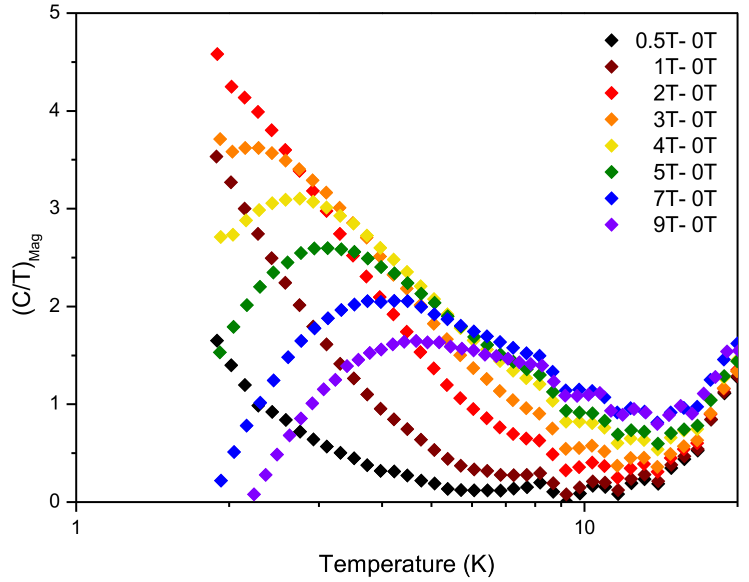

Figure S21. Temperature dependence of the magnetic component of C/T, after the subtraction of the $(C/T)_{0T}$, for YbGd-RPF-4.

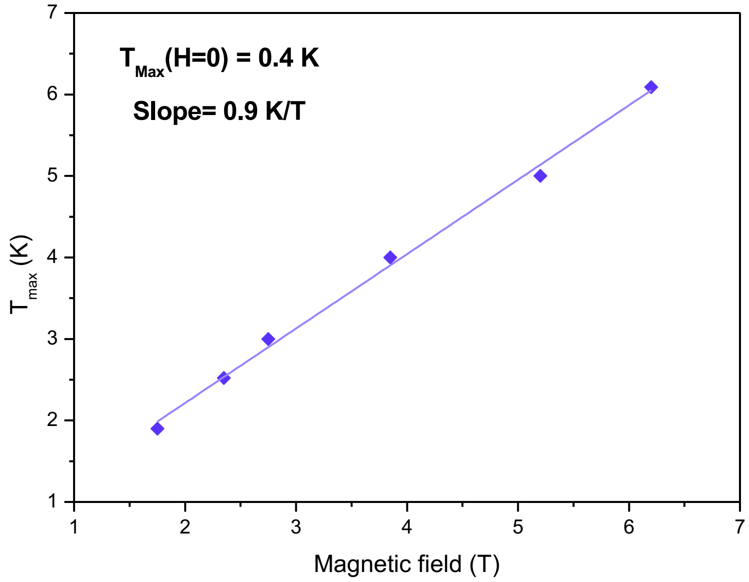

Figure S22. Magnetic Field dependence of the T_{Max}, for YbGd-RPF4, as obtained from the isothermal specific heat.

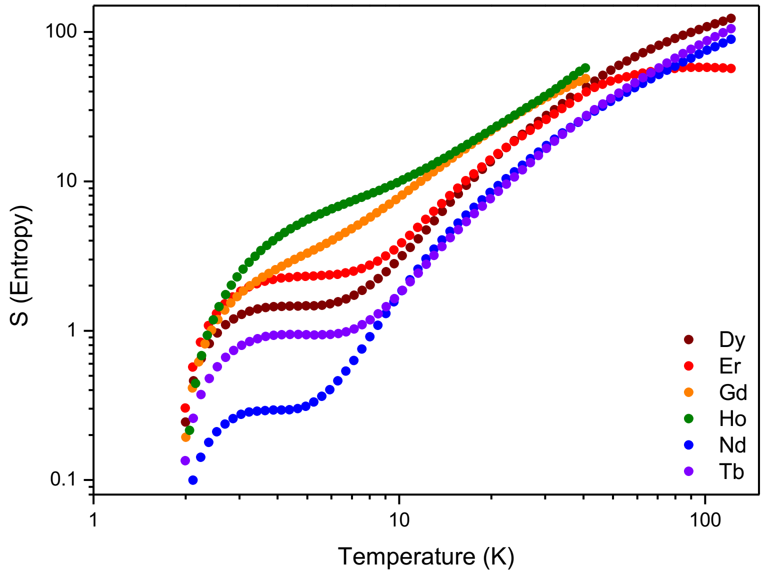

Figure S23. Magnetic entropy as a function of temperature, in logarithmic scale, for different rare earth ions of the RPF-4 family.

The following model was employed to fit the extracted magnetic heat capacity after removal of the La-RPF-4 matrix: a cubic background was employed to account for the remnants up to higher temperatures and a 2-level Schottky heat capacity was used to estimate the gap energy. The Schottky-model allows different degeneracies for the ground and excited levels, and this is not a straightforward issue here. The degeneracy of the ground and excited levels was chosen after analyzing various options for each sample in each field. Many samples could be analyzed with the simplest model: two non-degenerate levels (Ce-Ce, Gd-Gd, Ho-Ho, Nd-Nd, Tb-Tb)-RPF-4. However, Eu-RPF-4 could only be analyzed using double degenerate ground level. Nevertheless, although Er-RPF-4 must be analyzed using double degenerate ground level in intermediate fields, non-degenerate ground level gives similar gap energies in low and high fields. Both Yb-RPF-4 and Dy-RPF-4 could be best analyzed with double degenerate excited levels, although Dy-RPF-4 required non-degenerate levels in the highest field, whereas Yb-RPF-4 required non-degenerate excited level in the lowest field.

Furthermore, many samples showed a transition of the gap vs. field slope at intermediate magnetic fields. Additionally, the mixtures of YbDy-RPF-4 and YbGd-RPF-4 were also compared with the single-metal samples. YbDy-RPF-4 could be analyzed with non-degenerate levels, although neither Yb-RPF-4 nor Dy-RPF4 could be. YbGd-RPF-4 could be mostly analyzed with double-degenerate excited state, similar to Yb-RPF-4, although Gd-RPF-4 does not need this.

As an example, Fig. S24 illustrates the Schottky analysis of Er-RPF-4, since it can be analyzed using either non-degenerate or double-degenerate ground levels. The figure shows the Schottky-fit to the extracted ΔCp(T) excess or magnetic heat capacity data at a few magnetic fields. The Schottky model is based on the statistical physics of a few level system with thermal occupation, yielding[53]

$$C_{Sch}(T)=\frac{R}{T^2}\left[\frac{\sum \nu_i \Delta_i^2 e^{-\Delta_i/T}}{\sum \nu_i e^{-\Delta_i/T}}-\left(\frac{\sum \nu_i \Delta_i e^{-\Delta_i/T}}{\sum \nu_i e^{-\Delta_i/T}}\right)^2\right]$$

where Δ_i are the energy gaps (in K units), Δ_0=0 corresponds to the ground level. The model has only the gaps Δ_i as free parameters, once the number and degeneracy of levels, ν_i, is specified, leaving only one fitting parameter for 2-level systems. For 4f-electron systems at low temperatures it is often sufficient to consider only the first excited level together with the ground level. Such a two-level Schottky-system has a characteristic peak in the heat capacity. In our data, a free cubic background must also be included to account for the higher temperature evolution of the specific heat.

Schottky model analysis of low temperature specific heat with 2 nondegenerate levels and also with double degenerate ground-state level of the Er filled MOF.

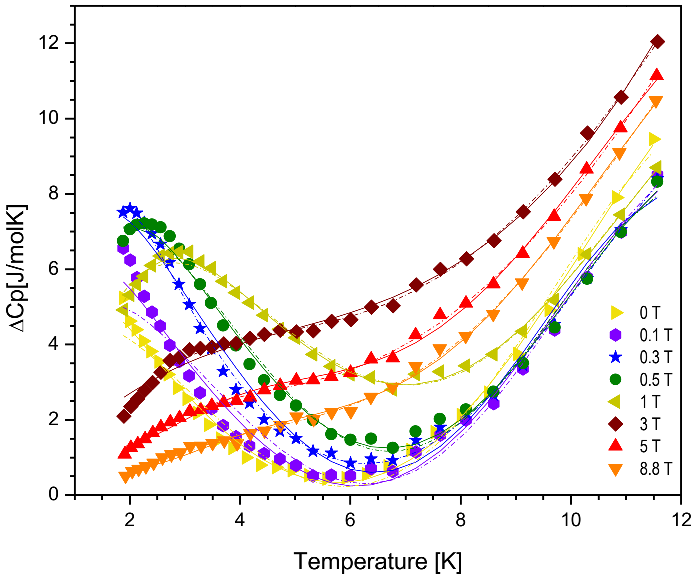

Figure S24. Temperature dependent excess or magnetic heat capacity of Er-RPF-4 in a few magnetic fields. The fits use non-degenerate (solid lines) and double-degenerate (dashed lines) ground levels.

Figure S25 shows the magnetic field dependent Schottky energy gaps for Ce-Ce, Gd-Gd, Ho-Ho, Nd-Nd, and Tb-Tb-RPF-4. All of these fillers could be analyzed with a 2-level non-degenerate Schottky model. The resulting Schottky Gap (H) dependence is also rather similar for Nd-Nd, Ce-Ce, Gd-Gd and Ho-Ho. The actual gap is 3-5 times larger for Ho-Ho in zero field, but the increase with H is the same for each. Tb-Tb is an outlier, the estimated gap does not show any field dependence above 1T, only below. At 0.5-1T it could be better analyzed with a double degenerate excited-level model, but this does not work at all in zero field or higher fields.

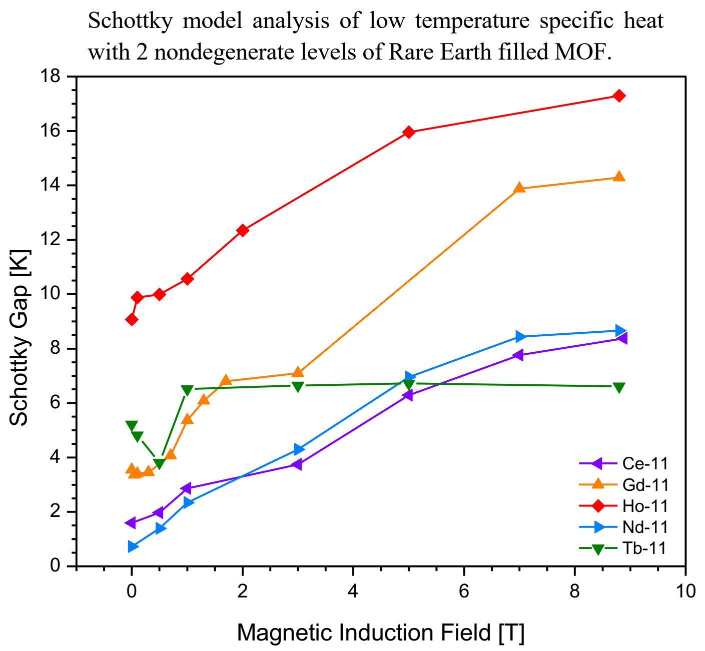

Figure S25. Magnetic field dependence of the estimated gap from a Schottky analysis with RE = Ce (purple triangles), Gd (orange triangles), Ho (red diamonds), Nd (blue triangles), Tb (green triangles).

Figure S26 shows the magnetic field dependent Schottky energy gaps for Er- and Eu-RPF-4. These fillers could not be adequately analyzed with a 2-level non-degenerate Schottky model. However, they could be analyzed with a 2-level model with double-degenerate ground level. Eu-Eu (purple diamonds) does not show much Gap vs. H dependence, just like Tb-Tb. Er-Er could be analyzed for any magnetic field with the double degenerate model (orange triangles), although the non-degenerate model also works to some extent (blue triangles), with worse fit, but similar Gap values. It does show a change in the slope around 1.5 T in the Gap (H) behavior.

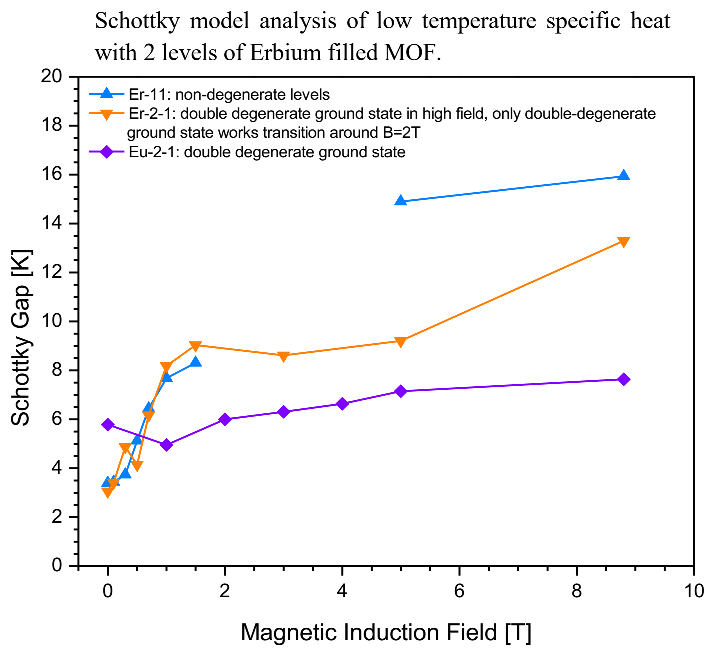

Figure S26. Magnetic field dependence of the estimated gap from a Schottky analysis with Eu-RPF-4 (purple diamonds) and Eu-RPF-4 (orange triangles for double degenerate ground state analysis and blue triangles for non-degenerate levels).

Figure S27 compares the magnetic field dependent Schottky energy gaps for multiple-filled YbDy-RPF-4 with those of single-filled Yb- and Dy-RPF-4. Both Yb -and Dy- filled MOF are best analyzed with a 2-level Schottky model having double-degenerate excited states.

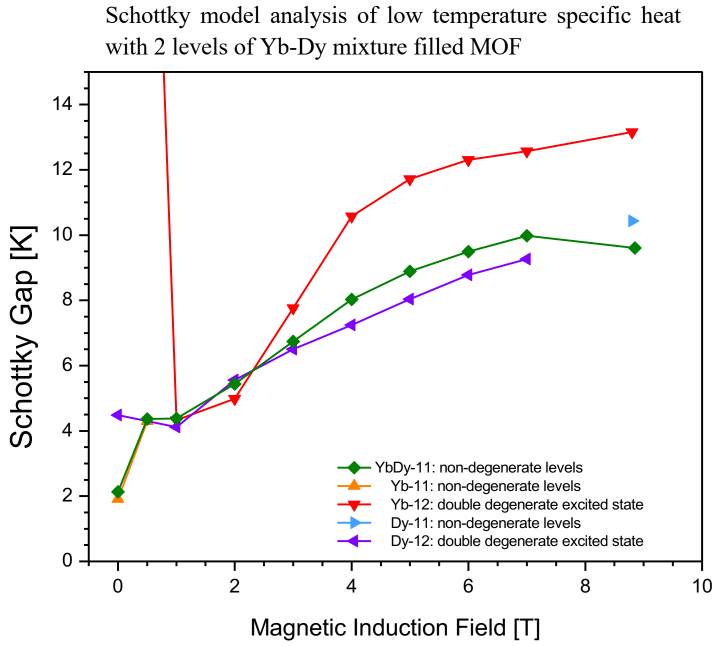

Figure S27. Magnetic field dependence of the estimated gap from a Schottky analysis with YbDy-RPF-4 (green diamonds) as compared to Yb- (orange and red triangles) and Dy-RPF-4 (blue and purple triangles).

This model works well for Dy- in all fields, except at 8.8T where only a non-degenerate model works. But the high field data are hard to fit anyway, for any compound. Dy-RPF-4 shows Gap (H) dependence similar to Ce-, Gd-RPF-4, with a bit less slope. Yb-RPF-4 could also be analyzed with this double-degenerate excited state model in all fields except very low fields, where the non-degenerate model works better. (the "vertical" red line reflects this, in low fields absurd Gap values are estimated with the excited-level model) Its Gap (H) dependence is stronger than that of Dy-RPF-4, more similar to Ce-, Gd-, Ho-RPF-4. However, the mixed YbDy-RPF-4 could only be analyzed with non-degenerate levels. Yet, the estimated gap energies practically coincide with those of Dy-RPF-4.

Figure S28 compares the magnetic field dependent Schottky energy gaps for multiple-filled YbGd-RPF-4 with those of single filled Gd- and Yb-RPF-4 (from previous figures). YbGd-RPF-4 can be analyzed with non-degenerate levels in high magnetic field, but in low fields double-degenerate excited levels are needed, yielding similar gap values in high field, somewhat similar to what must be done for Yb-RPF-4 (Fig. S26). However, Gd-RPF-4 can be analyzed in any magnetic field using non-degenerate levels (Fig. S24) The magnetic field dependence of the gap of the multiple-filled YbGd-RPF-4 is less pronounced than that of the single-filled compounds.

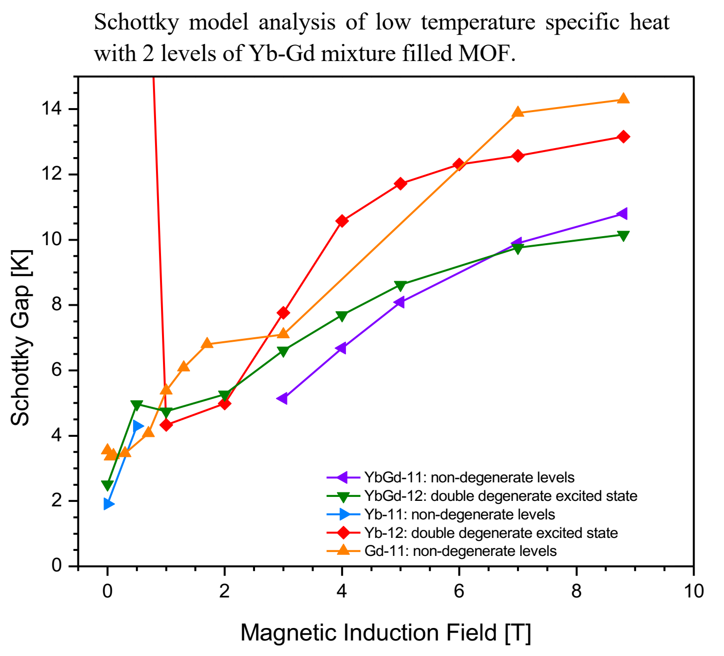

Figure S28. Magnetic field dependence of the estimated gap from a Schottky analysis with YbGd-RPF-4 (purple and green triangles), Gd-RPF-4 (orange triangles) and Yb-RPF-4 (blue triangles and red diamonds).

Computational details

Theoretical calculations by periodic density functional theory (DFT) were carried out using crystallographic data of each structure as stating point. Geometry and electronic structure were performed using Plane-wave density functional (PW-DF) calculations were done using the VASP package[1,2]. The total energies corresponding to the optimized geometries of all samples were calculated using the spin polarized version of the Perdew-Burke-Ernzerhof (PBE).[3] The effect of the core electrons on the valence electron density was described by the projector augmented wave (PAW) method.[4,5] The cutoff for the kinetic energy of the plane waves was set to 450 eV throughout, which after extensive tests proved to ensure a total energy convergence better than 10^{-5} eV.

1. G. Kresse and J. Furthmüller, *Computational Materials Science*, 1996, **6**, 15-50.
2. G. Kresse and J. Hafner, *Physical Review B*, 1993, **47**, 558-561.
3. JJ. P. Perdew, K. Burke, and M. Ernzerhof, Phys. Rev. Lett., 77, 3865 (1996).
4. G. Kresse and D. Joubert, *Physical Review B - Condensed Matter and Materials Physics*, 1999, **59**, 1758-1775.
5. P. E. Blöchl, *Physical Review B*, 1994, **50**, 17953-17979.,
